# Socioeconomic determinants of overweight and obesity among Mexican children and adolescents: Systematic review and meta‐analysis

**DOI:** 10.1111/obr.13926

**Published:** 2025-04-10

**Authors:** Magaly Aceves‐Martins, Yareni Yunuen Gutierrez‐Gómez, Carlos Francisco Moreno‐García

**Affiliations:** ^1^ The Rowett Institute of Nutrition and Health University of Aberdeen Aberdeen UK; ^2^ School of Medicine and Health Sciences Tecnologico de Monterrey Guadalajara Mexico; ^3^ School of Computing, Engineering and Technology Robert Gordon University Aberdeen Scotland, UK

**Keywords:** adolescents, children, Mexico, obesity, overweight, socioeconomic status

## Abstract

Socioeconomic status (SES) has widely been studied as a potential risk factor for obesity among children and adolescents. Nevertheless, SES determinants are rarely contextualized within a country's situation. This work aimed to identify SES factors associated with childhood and adolescent obesity in Mexico. Eleven scientific databases were searched, and 54 studies met the inclusion criteria. When measuring SES, 56% of the studies measured wealth, 50% living environment (urban vs rural areas), 44% parental education, 30% ethnic origin, 24% income or monetary measurements, 20% parental occupation and 18% the type of school participants attended. We found that Mexican children and adolescents were significantly more likely to have either overweight or obesity if they had a higher wealth (estimated through household characteristics) (OR 1.43, 95% CI 1.19, 1.72), lived in urban areas (OR 1.41, 95% CI 1.20, 1.66), identified as non‐Indigenous (OR 1.55, 95% CI 1.22, 1.96), had mothers with secondary school studies or higher (OR 1.44, 95% CI 1.14, 1.82), or mothers who were employed (OR 1.39, 95% CI 1.30, 1.48). Not all indicators of a higher SES (e.g., attending private school or not participating in a food provision program) were significantly associated with childhood overweight or obesity in Mexico. Furthermore, the evidence for other indicators, such as family structure, family size, household income, and monetary measures, remains uncertain. This work presents evidence of childhood obesity inequalities in Mexico.

## 1 INTRODUCTION

Socioeconomic status (SES) influences various lifestyles, including food access and physical activity patterns, affecting energy balance and eventually impacting nutritional status.^1^ When accounting for SES, various factors are comprised (e.g., income, education, occupational status, and access to resources). Nevertheless, measuring SES among young people can be complex and challenging as, unlike adults, children's SES is typically inferred from their parents' SES measurements or the environment they live in. Usually, SES estimators for children and adolescents include parental characteristics (e.g., parental education level or employment status), household measures (e.g., household income), or broader indicators at a setting level (e.g., neighborhood or school characteristics).^2^, ^3^, ^4^

SES has been extensively studied as a potential risk factor for obesity in children and adolescents.^1^, ^2^, ^3^, ^4^, ^5^, ^6^, ^7^, ^8^ Overall, studies are inconsistent in terms of the direction and strength of these associations. Some studies have suggested that the association between SES and obesity in young populations is only significant when considering their age, sex, or ethnicity.^1^, ^2^, ^4^ Some others suggest that the likelihood of overweight and obesity is higher in young people with lower SES compared to those with higher SES, but only in high‐income countries.^3^ Nevertheless, evidence from low‐ and middle‐income countries is often underappreciated in meta‐analyses of this topic, as only a few or no studies are included.^3^, ^8^ Moreover, few studies consider the cultural and geographic variability of SES factors, leading to a lack of contextualization within a country's circumstances.

Mexico is classified as an upper‐middle‐income country that has made tremendous progress over the last decades in improving its citizens' quality of life.^9^, ^10^ Notwithstanding, Mexico continues to struggle in various dimensions of well‐being and is currently facing several public health challenges, including having one of the highest rates of childhood and adolescent obesity in the world.^11^ The uprising trends have been reported over the last decades, with the latest figures in 2020 showing that the prevalence of overweight and obesity for school‐age children was 19.6% and 18.6%, respectively, and for adolescents, 26.8% and 17%, respectively.^12^

Several SES factors significantly impact Mexico's development, including poverty rates, economic growth and income inequality, education, health care, and access to essential services (e.g., clean water or electricity).^13^ Additionally, SES factors shape individuals' access to resources, which in turn influence their behaviors and health outcomes.^14^ Recognizing the role of SES in childhood obesity in Mexico is essential for relevant stakeholders, such as public health authorities, researchers, and community members, to design and implement effective, long‐term strategies. The “Childhood and adolescent Obesity in MexicO: evidence, challenges and opportunities” (COMO) Project intends to synthesize and use data to comprehend the extent, nature, effects, and costs of childhood and adolescent obesity in Mexico.^15^, ^16^, ^17^, ^18^, ^19^, ^20^ This systematic review and meta‐analysis are part of the COMO project and aim to identify SES factors related to childhood and adolescent obesity in Mexico.

## 2 MATERIAL AND METHODS

This project's systematic review was registered in The International Prospective Register of Systematic Reviews (PROSPERO Registration CRD42019154132)^21^ and followed the Preferred Reporting Items for Systematic Reviews and Meta‐analyses (PRISMA) guidelines.^22^ The research question and inclusion/exclusion criteria were defined following the Population, Exposure, Comparison, Outcome, and Study design (PECOS) framework for quantitative synthesis.

### 2.1 Search strategy

A sensitive search was developed to include index terms, free‐text words, abbreviations, and synonyms to combine the key concepts for this review (Appendix 1). The databases searched included EMBASE, MEDLINE, LILACS, CINAHL, Global Health Library, ERIC, PsycINFO, ScienceDirect, Scopus, AGRICOLA, and SciELO Citation Index. Whenever possible, searches were done in Spanish to capture relevant references. In addition, the search engine Google Scholar and the COMO project database were used. The COMO project database currently includes over 1200 references relevant to childhood and adolescent obesity in Mexico. These references have been collected from indexed, non‐indexed, and gray literature sources since 2020 and encompass evidence dating back to the early 1980s.^15^ In addition, reference lists of included papers were examined for additional publications. This review considered full‐text papers and abstracts in English, Spanish, or Portuguese from studies published from 1995 onward, and searches were done in June 2024.

### 2.2 Selection criteria

Based on the PECOS framework, the eligibility criteria were as follows:

*Population*: Children and adolescents from 0 to 18 years old of any ethnicity or sex living in Mexico were included. Studies that involved parents or caregivers were included only if the outcomes were measured in children or adolescents. Mexican children living in different countries were excluded to better conceptualize the obesity problem within the country, avoiding confounding information inherent to the migration phenomena.
*Exposure*: Studies that included an analysis of at least one SES estimator associated with the outcome (i.e., overweight or obesity) were included. According to the American Psychological Association, SES encompasses income, educational attainment, occupational prestige, and subjective social status and class perceptions.^23^ Studies were included if these considered any income or social status indicators from families or households (e.g., household income, parental education level or parental employment status) or at a setting level (e.g., neighborhood location or school characteristics). Studies were excluded if participants were recruited from a specific SES category (e.g., participants only from low‐income neighborhoods), as this review was meant to compare the outcome across different SES categories. Also, studies analyzing individual characteristics of children (e.g., age or sex) were not considered in this review.
*Comparator*: Any or none
*Outcomes*: Studies were included in this review if they reported quantitative estimates (e.g., prevalence, odds ratio [OR], means) of overweight or obesity from participants through weight‐related outcomes (e.g., Body Mass Index [BMI], BMI z‐score). Studies had to provide the nutritional status of participants based on a national or international reference to be included. Studies using weight‐related outcomes as a continuous variable (without classifying participants according to their nutritional status) were excluded from this review. Studies focusing exclusively on any form of underweight were excluded from this review, as these were out of the scope.
*Study design*: Observational studies.

### 2.3 Data selection

Two reviewers (MA‐M, CFM‐G) screened titles, abstracts, and relevant full texts. One reviewer (MA‐M) extracted data from the studies and checked 10% by a second reviewer for consistency (CFM‐G). A third author (Y.Y.G.‐G.) was contacted in case of any disagreement. A data extraction form was created following the PECOS framework, which included relevant data from the included studies, such as population characteristics (e.g., sample size, target population, mean age, sex distribution), study design; setting characteristics (e.g., city, Mexican state, recruitment location); exposure (any SES indicator relevant to the household or environment of the included participant), outcome (e.g., BMI and any other anthropometric or adiposity measurement considered).

### 2.4 Data synthesis

The data extracted from the included studies were synthesized narratively, and the main characteristics were tabulated. As has been reported previously,^16^, ^24^ the nutritional status of young people might vary depending on the age of the references used to categorize BMI. Usually, four BMI categories are used when classifying children and adolescents, including “underweight,” “normal weight,” “at risk of overweight,” and “overweight.” Some other references use “underweight,” “normal weight,” “overweight,” and “obesity.” For the synthesis purposes of the current work, the categories “at risk of overweight” and “overweight” were unified. Moreover, the categories “overweight” and “obesity” will consider children and adolescents within the two highest BMI categories, regardless of the anthropometric reference used across studies.

Relevant SES indicators of participants, including measurements related to wealth, income, education, occupational prestige, and subjective social status, relevant to the children, their parents, household, or living/studying area, were categorized into the following main groups: wealth, income, living environment (urban vs. rural), education, ethnicity, and parental occupation or employment. The results section of this review outlines how each SES indicator or category was standardized and grouped for analytical purposes.

### 2.5 Risk of bias assessment

The JBI (formerly known as Joanna Briggs Institute) critical appraisal tool for cross‐sectional studies was used to assess the quality of the included studies.^25^ This tool assessed the methodological quality of the included studies by evaluating how well each study addressed potential biases. Within the evaluation, eight critical items were considered: explicit definition of inclusion and exclusion criteria; study participants and setting details; identification of confounding factors; strategies to deal with confounding factors; SES measurements; standard criteria used for measurement of the condition; outcome measurement validity and reliability; and statistical analysis appropriateness. Articles were not included or excluded based on their quality, but the appraisal results were considered in the synthesis process.

### 2.6 Data analysis

Whenever possible, the Odds Ratio (OR) for overweight and obesity were calculated. If raw data to estimate ORs was unavailable, but an unadjusted OR was provided, this was considered in the analysis. To showcase those participants with higher BMIs and increase the sample size in meta‐analyses, as a primary analysis, participants with overweight and obesity were pooled and compared with data on lower BMIs (normal weight); if the data allowed it, the “underweight category” was excluded from the analysis. As a secondary analysis, meta‐analyses of specific BMI (overweight or obesity) and/or SES categories were conducted, and results are provided in Appendix 2. Considering that all the papers included were observational studies, a DerSimonian and Laird method was used to construct a random‐effects model to account for the heterogeneity within and between studies.^26^ All results were reported with OR and 95% Confidence Intervals (CI), and the main results are presented in forest plots. The analysis was performed with R statistical software using the library “*meta*” and “*metafor*”.

## 3 RESULTS

**FIGURE 1:**
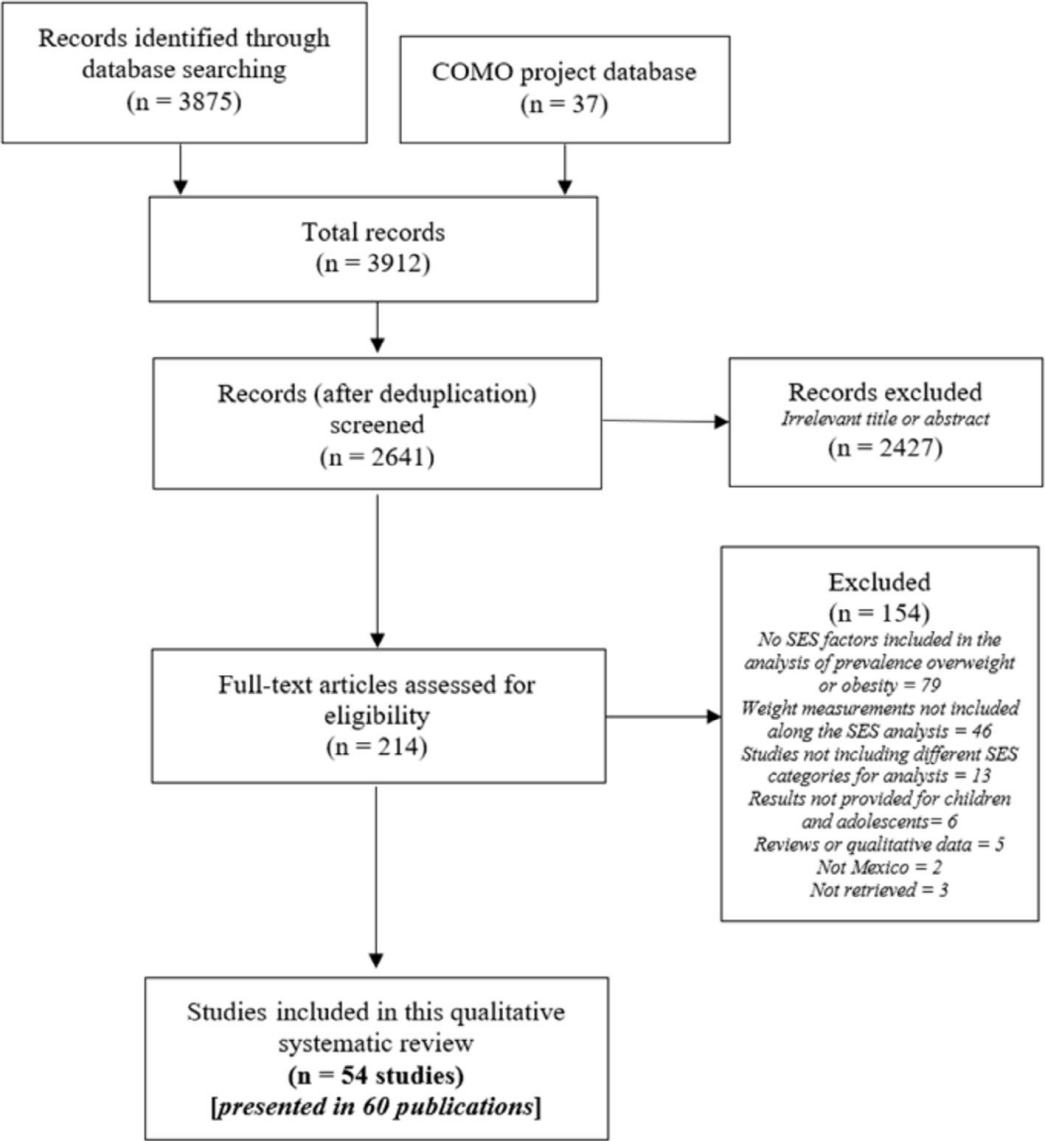
PRISMA flowchart.

After the systematic search, 2641 unique references were identified, and 212 were retrieved for full‐text review. Of these, 54 studies (presented in 60 references)^27^, ^28^, ^29^, ^30^, ^31^, ^32^, ^33^, ^34^, ^35^, ^36^, ^37^, ^38^, ^39^, ^40^, ^41^, ^42^, ^43^, ^44^, ^45^, ^46^, ^47^, ^48^, ^49^, ^50^, ^51^, ^52^, ^53^, ^54^, ^55^, ^56^, ^57^, ^58^, ^59^, ^60^, ^61^, ^62^, ^63^, ^64^, ^65^, ^66^, ^67^, ^68^, ^69^, ^70^, ^71^, ^72^, ^73^, ^74^, ^75^, ^76^, ^77^, ^78^, ^79^, ^80^, ^81^, ^82^, ^83^, ^84^, ^85^ met the inclusion criteria (Figure 1). Seven references^50^, ^63^, ^70^, ^71^, ^79^, ^80^ were abstracts, and the rest full text. Seventeen studies^27^, ^32^, ^36^, ^37^, ^38^, ^40^, ^41^, ^43^, ^44^, ^45^, ^49^, ^51^, ^52^, ^54^, ^58^, ^60^, ^75^, ^76^ included large nationwide samples, such as the National Survey of Nutrition and Health (ENSANUT). Most of the studies included a cross‐sectional design, except two^53^, ^80^ which conducted a case–control design and one longitudinal.^38^ The design of one of the studies presented in an abstract was unclear.^63^ The sample ranged from 72 participants^35^ up to 10,528,676, which included data from the National Weight and Height Registry.^27^ All the studies included males and females except for one^60^ with only females. (Table 1).

**TABLE 1:** Main characteristics of included studies.

*Study characteristics*	*Population characteristics*	*Outcome measurements*	*SES factors measured*								
*Study ID linked study (if any) study design study location*	*Sample size* *Female (%)* *Age other characteristics*	*Overweight and obesity prevalence* *Weight recall method* *Refeence used to classify participant's nutritional status*	Wealth	Living setting	Parental education	Family structure and size	Ethnic origin	Income	Parental employment	Type of school	Other factors
**Ávila‐Curiel 2021** ^27^ National Weight and Height Registry 2016 Cross‐sectional study National sample	n = 10,528,676 F=NR 6–12 years old The study population was based on all schools in the primary education system of the Ministry of Public Education	34.4% Trained personnel WHO	✓	✓	×	×	×	●	×	✓	×
**Bacardí‐Gascón 2007** ^28^ Cross‐sectional study Ensenada, Baja California	n = 967 F = 51% 6–12 years old Public and private schools were randomly chosen from Ensenada.	45% Trained personnel CDC	×	×	×	×	×	×	×	✓	×
**Bacardí‐Gascón 2009** ^29^ Cross‐sectional study Tijuana, Baja California	n = 1684 F = 49% 6–14 years	53.5% Trained personnel CDC	×	×	×	×	×	×	×	✓	×
**Basaldua 2008** ^30^ Cross‐sectional study Magdalena de Kino, Sonora	n = 551 F = 50% 6–12 years Participants were recruited from 2 public institutions in an urban region of Sonora. All the children were of Latin‐American ethnicity, and their family had an annual income of USD < 15,000.	37.6% Trained personnel IOTF	×	×	×	✓	×	×	×	×	×
**Batis 2020** ^31^, ^32^ ENSANUT 2012Cross‐sectional study National sample	n = Children < 5 years old: 10658, Women 11–19 years old: 8044. F=Children > 5 years old: 49.8%, Women 11–19 years old: 100%. Children <5 years old, Women 11–19 years old.	Children <5 years: 9.7% Women 11–19 years: 36.0% Trained personnel WHO	✓	✓	✓	×	✓	×	×	×	×
**Benítez‐Hernández 2014** ^33^, ^34^ Cross‐sectional study 50% from Agua Zarca, Guachochi, and 50% from Chihuahua City, Chihuahua	n = 100 F = 54% 6–14 years Tarahumaras Indians, with 50% recruited in rural areas and 50% in urban areas.	22% Trained personnel IOTF	×	✓	×	×	●	×	×	×	×
**Bernabeu‐Justes 2019** ^35^ Cross‐sectional study Suchitlán, Cofradía de Suchitlán and Zacualpan, Colima	n = 72 F = 52% 1–5 years	11.1% Trained personnel WHO	×	×	●	✓	×	●	×	×	×
**Bojorquez 2018** ^36^ National Survey of Drug Use in Students (ENCODE, as per its acronym in Spanish) Cross‐sectional study National sample	n = 28,266 F = 50% 13–15 years	Males: 41.4% Females: 32.5% Self‐reported WHO	×	✓	✓	×	✓	●	×	×	×
**Bonvecchio 2009** ^37^ National Survey 1990 ENSANUT 1999 ENSANUT 2007 Cross‐sectional study National sample	n = 62,494 children: 9682 from 1988, 19,353 from 1999 and 33,459 from the 2006 F = 50% 2–18 years	26.3% Trained personnel IOTF	✓	✓	×	×	✓	×	×	×	×
**Brambila‐Paz 2022** ^38^ Mexican Family Life Survey Longitudinal Study National sample	n = 3202 F = 48% 5–12 years (at baseline) Participants were recruited in 2002 and followed up in 2009–2012. The first survey round was conducted in 147 urban and rural communities nationwide.	3.1% Trained personnel WHO	✓	×	●	●	×	×	●	×	✓
**Brewis 2003** ^39^ Cross‐sectional study Xalapa, Veracruz	n = 219 F = 50% 6–12 years Participants were from the middle‐class population who attended a single‐state public elementary school.	24.2% (>95th percentile compared to gender‐specific CDC 2000 references) or 50.5% (>85th percentile) Trained personnel CDC	✓	✓	●	✓	×	●	✓	×	×
**Campos 2021** ^40^ ENSANUT 2012 Cross‐sectional study National sample	n = 2089 F = 44% 6–35 months	58.5% Trained personnel WHO	✓	✓	✓	✓	✓	×	✓	×	×
**Cárdenas‐Villarreal 2023** ^41^ ENSANUT 2012–2020 Cross‐sectional study National sample	n = 6719 (3854 in 2012; 1096 in 2016; 1234 in 2018–2019; and 535 in 2020) F=NR <24 months	In 2012:7.8% In 2020:10.3% Trained personnel WHO	✓	✓	×	×	✓	×	×	×	×
**Cauich‐Viñas 2019** ^42^ Cross‐sectional study Merida and Motul, Yucatan	n = 260 F=NR 6–12 years Mother and child dyads were Mayan and randomly recruited from primary schools of the participant cities.	43.9% Trained personnel WHO	×	×	✓	✓	●	×	×	×	×
**Cuevas‐Nasu 2009** ^43^ ENSANUT 2006 Cross‐sectional study National sample	n = 15,003 F = 50% 5–11 years	26.3% Trained personnel IOTF	✓	✓	×	×	×	×	×	×	✓
**Cuevas‐Nasu 2017** ^44^ ENSANUT 2016 Cross‐sectional study National sample	n = 1993 F = 49% <5 years	5.8% Trained personnel WHO	×	✓	×	×	×	×	×	×	×
**Del Monte‐Vega 2021** ^45^ National Registry of Weight and Height Cross‐sectional study National sample	n = 2,529,091 F=NR 6–12 years Data since 2015 was recorded; however, due to the high risk of overlap, only the 2018–2019 was used.	33.8% Trained personnel WHO	✓	✓	×	×	×	×	×	×	×
**Fernald 2007** ^46^ National Social Welfare Survey Cross‐sectional study Rural towns within Guerrero, Hidalgo, Michoacán, Puebla, Querétaro, San Luis Potosí and Veracruz	n = 8228 F = 49% 2–6 years Low‐income households within low‐income (mean daily per capita income was $2US) communities were poor (income <20th percentile) and rural (defined as towns with <2500 inhabitants). All households received the benefits of a social program for a minimum of 3 years	25.0% Trained personnel CDC	✓	×	✓	✓	✓	×	×	×	✓
**Flores 2019** ^47^ Cross‐sectional study Cuernavaca, Morelos	n = 181 F = 48% 11–18 years	76.2% Clinical Staff WHO	×	×	✓	×	×	×	×	×	×
**Flores‐Guillen 2023** ^48^ Birth cohort study, born in three public hospitals in Chiapas in 2003 Cross‐sectional study From 14 municipalities in the Tzotzil‐Tzeltal and Selva regions, Chiapas	n = 253 F = 48% Age not reported The sample of adolescents from a birth cohort study was born in three public hospitals in 2003.	Overweight/Obesity (BMI > 1 z‐score) 28.9% Abdominal obesity (≥ 80 cm in girls and ≥ 90 cm in boys) 14.6% Trained personnel WHO	●	✓	✓	×	●	×	×	×	●
**Flores‐Huerta 2012** ^49^ Cross‐sectional study National sample	n = 8328 F=NR <2 years	6.3% Trained personnel WHO	×	✓	×	×	×	×	×	×	×
**Galvan 2011 (A)** ^50^ Cross‐sectional study Two municipalities, Hidalgo	n = 1400 F=NR 7–12 years	NR Unclear WHO	×	×	×	×	×	×	×	✓	×
**García‐Chávez 2020** ^51^ ENSANUT 2012 Cross‐sectional study National sample	n = 2751 F = 48% 5–11 years NR	32.1% Trained personnel WHO	✓	✓	✓	×	×	×	×	×	×
**García‐Guerra 2012 (A)** ^52^ ENSANUT 2006 Cross‐sectional study National sample	n = NR F=NR 2–5 years	Only reported for Girls: Low inequality index: 29.1%, Medium inequality index: 22.6% and High inequality index: 27.5% Trained personnel, WHO	×	×	×	×	×	✓	×	×	×
**González‐Rico 2012** ^53^ Case–control study Jalisco	n = 452 F = 46% 6–9 years Participants were from an entitled population from the Family Medicine Unit 3 of the Mexican Institute of Social Security	34.5% Clinical Staff NR	×	×	✓	×	×	●	●	×	×
**Hernandez 2003** ^54^ National Nutrition Survey 1999 (NNS‐1999) ‐ National Household Sampling Frame (Marco Muestral Nacional de Hogares) of the National Institute of Statistics, Geography, and Informatics (Instituto Nacional de Estadística, Geografía e Informática, INEGI) Cross‐sectional study National sample	n = 10,901 F = 50% 5–11 years	19.5% Trained personnel WHO	✓	✓	✓	×	✓	×	×	×	×
**Jimenez‐Cruz 2010** ^55^ Cross‐sectional study Tijuana, Tuxtla y Reynosa, Baja California, Chiapas y Tamaulipas	n = 810 F = 49% 5–24 months Participants were defined as belonging to “low‐income families”. This was defined as per recruitment method, where participants were recruited from public clinics where patients usually come from the lowest income levels.	41.9% Trained personnel WHO	×	×	✓	×	×	✓	×	×	×
**López‐Morales 2016** ^56^ Cross‐sectional study Sonora, Cd. Obregón	n = 120 F = 50% 15–18 years All recruited from public clinics	50% Clinical Staff CDC	×	✓	×	✓	×	×	●	×	×
**Malina 2009** ^57^ Cross‐sectional study Community located south and west of Oaxaca de Juarez across the Atoyac River, Oaxaca.	n = 781 F = 47% 6–13 years Children were enrolled in the same school in 1972 and 2000, and the recruitment site is described as primarily a Mixtec town.	Reported by year, gender and SES (based on parental occupation) level: Boys Low SES 2000:10% Low Middle SES 2000:19% Middle SES 2000:14% Girls Low SES 2000:11% Low Middle SES 2000:16% Middle SES 2000:22% Trained personnel CDC	×	✓	×	●	×	×	✓	×	×
**Martínez‐Espinosa 2018** ^58^ ENSANUT 2012 Cross‐sectional study National sample	n = 14,718 F = 49% 5–17 years	35.7% Trained personnel WHO	✓	✓	✓	✓	✓	×	✓	×	×
**Martinez‐Navarro 2022** ^59^ Cross‐sectional study Mexico City	n = 210 F = 50% 6–10 years	50.9% Trained personnel WHO	✓	×	✓	×	×	×	×	✓	×
**Medina‐Zacarías 2020** ^60^ ENSANUT 2016 Cross‐sectional study National sample	n = 1072 F = 100% 12–19 years	36.6% Trained personnel WHO	✓	✓	×	✓	✓	×	×	×	✓
**Mendez 2016** ^61^ Cross‐sectional study Merida, Yucatan	n = 3243 F = 53% 5–11 years	50.8% Trained personnel WHO	×	×	×	×	✓	✓	×	×	×
**Miranda‐Rios 2017** ^62^ Cross‐sectional study Arandas, Jalisco	n = 192 F = 42% 5–12 years Participants recruited from public schools	27.6% Trained personnel WHO	✓	×	✓	✓	×	●	✓	×	×
**Morales‐Ruan 2015 (A)** ^63^ Study design is unclear, but observational San Luis Potosi	n = 403 F=NR School‐aged children	PDE group: 20.4% Control group:16% Unclear Unclear	✓	×	×	×	×	×	×	×	✓
**Mota‐Sanhua 2008** ^64^ Cross‐sectional study Mexico City	n = 486 F = 43% 12–16 years Participants were recruited from a public secondary school.	35.4% Clinical Staff CDC	×	×	✓	✓	×	×	✓	×	×
**Ortiz‐Hernández 2005** ^65^ Cross‐sectional study Mexico City	n = 972 F = 49% 7‐13yeaers	7.9% Trained personnel CDC	✓	×	×	×	×	×	×	×	×
**Ortiz‐Hernández 2007** ^66^ Cross‐sectional study Mexico City	n = 768 F = 46% 9–15 years old Children were recruited from four public and two private schools. Those that were public were in marginalized neighborhoods.	9.3% Unclear CDC	✓	×	×	×	×	×	×	×	×
**Peña‐Reyes 2010** ^67^ Cross‐sectional study 248 localities in Oaxaca, Oaxaca	n = 11,454 F = 45% 6–14 years Participants were from bilingual schools for Indigenous children	NR Trained personnel WHO	✓	✓	●	×	●	●	×	×	×
**Ramírez Serrano 2021** ^68^ Cross‐sectional study Colima City, Colima	n = 120 F = 52% 5–11 years Participants recruited from a public clinic.	43.7% Clinical Staff WHO	✓	×	×	✓	×	✓	×	×	×
**Rivera‐Ochoa 2020** ^69^ The Healthy Lifestyle in Mexico by Nutrition in Adolescence (HELENA) MEX Cross‐sectional study Guadalajara, Santa Maria de los angeles, Huejucar, Colotlan, Villaguerrero, Jalisco	n = 469 F = 55% 14–16 years	33.2% Trained personnel WHO	×	✓	×	×	×	×	×	×	×
**Romano 2012 (A)** ^70^ Cross‐sectional study Queretaro, Queretaro	n = 1102 F = 49% 5–12 years	44% Unclear Unclear	×	×	✓	×	×	×	×	✓	×
**Romero‐Velarde 2009 (A)** ^71^ Cross‐sectional study Guadalajara, Jalisco	n = 328 F=NR Age NR	NR Unclear Unclear	✓	×	×	×	×	×	×	×	×
**Rosas 2011** ^72^ Proyecto Mariposa Cross‐sectional study Guanajuato, Jalisco and Michoacan	n = 316 F = 53% 5‐year‐olds Beneficiaries of a social welfare program	15% Trained personnel CDC	✓	×	✓	✓	×	×	✓	×	×
**Salazar‐Martinez 2006** ^73^ Cross‐sectional study Morelos	n = 10,537 F = 65% 11‐19 years old	29% Trained personnel CDC	✓	✓	×	×	×	●	×	×	×
**Shamah‐Levy 2019** ^74^, ^75^, ^76^ ENSANUT 2012 and 2018 Cross‐sectional study National sample	n = 2012: 34261; 2018: 27120. F=NR. 0–19 years	Reported by different characteristics and per year of the survey. Trained personnel WHO	✓	✓	×	×	✓	×	×	×	✓
**Torres‐González 2019** ^77^ Cross‐sectional study Durango, Durango	n = 24,600 F = 20% 6–11 years	35.6% Trained personnel WHO	×	✓	×	×	×	×	×	×	×
**Ullmann 2011** ^78^ Encuesta Nacional de Salud 2000 Cross‐sectional study Mexico City	n = 10,069 F=NR. 10–18 years The sample was restricted to adolescents cohabiting with both parents.	8.2% (Obesity only) Unclear IOTF	✓	✓	✓	●	×	×	×	×	×
**Vasquez‐Garibay 2011 (A)** ^79^, ^80^ Case–control study Jalisco, Guadalajara	n = 452 F=NR 6–9 years Participants recruited from public Family Medicine Units	NR Clinical Staff Unclear	×	×	✓	✓	×	✓	✓	×	×
**Vázquez‐Nava 2013** ^81^ Cross‐sectional study Tampico‐Madero Altamira area, Tamaulipas	n = 897 F = 47% 6–12 years The children who participated in this study were recruited from eight different public and private elementary schools located in the urban area	40.7% Trained personnel CDC	×	×	✓	✓	×	×	✓	×	×
**Veile 2022** ^82^ Cross‐sectional study Merida, Motul and Puuc region, Yucatan	n = 138 F=NR 6‐year‐olds Maya children	Rural: 7.5% prevalence of OW (no OB) Urban: 45.8% prevalence of OW + OB Trained personnel WHO	×	✓	×	×	●	×	×	×	×
**Velasco‐Martínez 2009** ^83^ Cross‐sectional study Tuxtla Gutiérrez, Chiapas	n = 259 F = 58% 12–15 years.	32% Trained personnel CDC	×	×	×	×	×	×	×	✓	×
**Villa‐Caballero 2006** ^84^ Cross‐sectional study Tijuana, Baja California, Chiapas y Tamaulipas	n = 1172 F = 49% 6–13 years	41.2% Trained personnel CDC	✓	×	×	×	×	×	×	✓	×
**Walker‐Pacheco 2011 (A)** ^85^ Cross‐sectional study Guadalajara and Tierranueva, Guadalajara	n = 210 F=NR 2–12 years	Urban: 32% Rural: 25% Unclear CDC	×	✓	×	×	×	×	×	×	×

Abstract only (A), SES factor considered in the study (✓), SES factor not considered in the study (×), SES factor recall as part of the general characteristics of the participants but not analyzed regarding the overweight or obesity prevalence (●), World Health Organisation (WHO), Centres for Disease Control and Prevention (CDC), US dollars (USD), International Obesity Task Force (IOTF), Not Reported (NR).

The anthropometric variables were collected by different means, with most of the studies collecting data through trained or clinical staff and using the CDC or WHO reference for categorization. The prevalence of overweight and obesity varied across studies, from 3.1%^38^ to 76.2%^47^. When measuring SES, the frequently most used proxy was wealth, including measurements based on household characteristics and goods ownership (56%), followed by living environment (urban vs rural areas) (50%), parental education (44%), ethnic origin (30%), income or monetary measurements (24%), parental occupation or employment (20%), or type of school (18%). A couple of other SES estimators were also identified and described below. All included studies investigated at least one SES variable and its association with the prevalence of overweight or obesity among Mexican children and adolescents. Some studies included more than one SES variable; however, in some cases, some SES characteristics were recalled and reported as part of participants' characteristics but were not included in the analysis of overweight or obesity. Full details of how these proxies were recalled across studies can be seen in Appendix 2. The results presented in this section are ordered according to the proxies where more evidence was found to the least.

### 3.1 Measurements of wealth

Twenty‐eight studies^27^, ^31^, ^32^, ^37^, ^38^, ^39^, ^40^, ^41^, ^43^, ^45^, ^48^, ^51^, ^54^, ^58^, ^59^, ^60^, ^62^, ^63^, ^65^, ^66^, ^67^, ^68^, ^71^, ^72^, ^73^, ^75^, ^76^, ^78^, ^84^ used household wealth measurements or neighborhood marginalization as proxies for SES. Most of these studies focused on household wealth based on housing characteristics and assets, except for six studies^27^, ^45^, ^67^, ^68^, ^71^, ^84^ which considered the degree of marginalization (including wealth indicators) of the area of residency or the location of the school that participants attended. All 28 studies, except for one^48^, studied wealth and marginalization and its association with overweight or obesity.

From those studies measuring household wealth, most^31^, ^32^, ^37^, ^38^, ^40^, ^41^, ^43^, ^51^, ^54^, ^58^, ^59^, ^60^, ^72^, ^73^, ^75^, ^76^, ^78^ used a component analysis, including different household characteristics, housing quality materials on the structure (e.g., floor, walls and roof), public services (e.g., public sanitary sewer system, public water network or electricity availability) and assets ownership (e.g., motor vehicle, television, fridges). Two studies^48^, ^65^ measured only household assets, and in two other studies^63^, ^71^ it was unclear how wealth was recorded. Six studies evaluated the degree of overcrowding (i.e., the number of people per room, which differs from the total number of family members, synthesized below in “Family structure and size”).^38^, ^39^, ^62^, ^65^, ^66^, ^67^ Three studies^27^, ^45^, ^68^ used the degree of marginalization provided by the National Population Council, which considers housing services, security perception in the neighborhood, education, and monetary income of the areas where the household is located. Three studies considered wealth or characteristics of neighborhoods or municipalities for the analysis^45^, ^67^, ^84^. One study^68^ considered the type of household, the perception of security, and the distance to the closest park to indicate wealth. One abstract^71^ reported using “school social class” as a variable, but how this was classified was unclear.

Overall, most studies found that overweight and obesity were more prevalent among children and adolescents from wealthier households, characterized by better household conditions and higher possession of goods.^37^, ^39^, ^40^, ^43^, ^45^, ^46^, ^51^, ^54^, ^58^, ^59^, ^60^, ^62^, ^63^, ^67^, ^72^, ^73^, ^75^, ^76^, ^78^, ^79^ Only one study^68^ that included school‐aged children recruited from a public family clinic found that children who lived in a neighborhood with a high or very high degree of marginalization had a higher prevalence of overweight than those who lived medium to a very low degree of marginalization neighborhoods (p = 0.022). One study^41^ that included national data from children 0–23 months reported an insignificant pattern of overweight prevalence based on wealth. However, this same study reported that from 2012 to 2020, the prevalence of overweight and obesity almost doubled in individuals with lower wealth.

**FIGURE 2:**
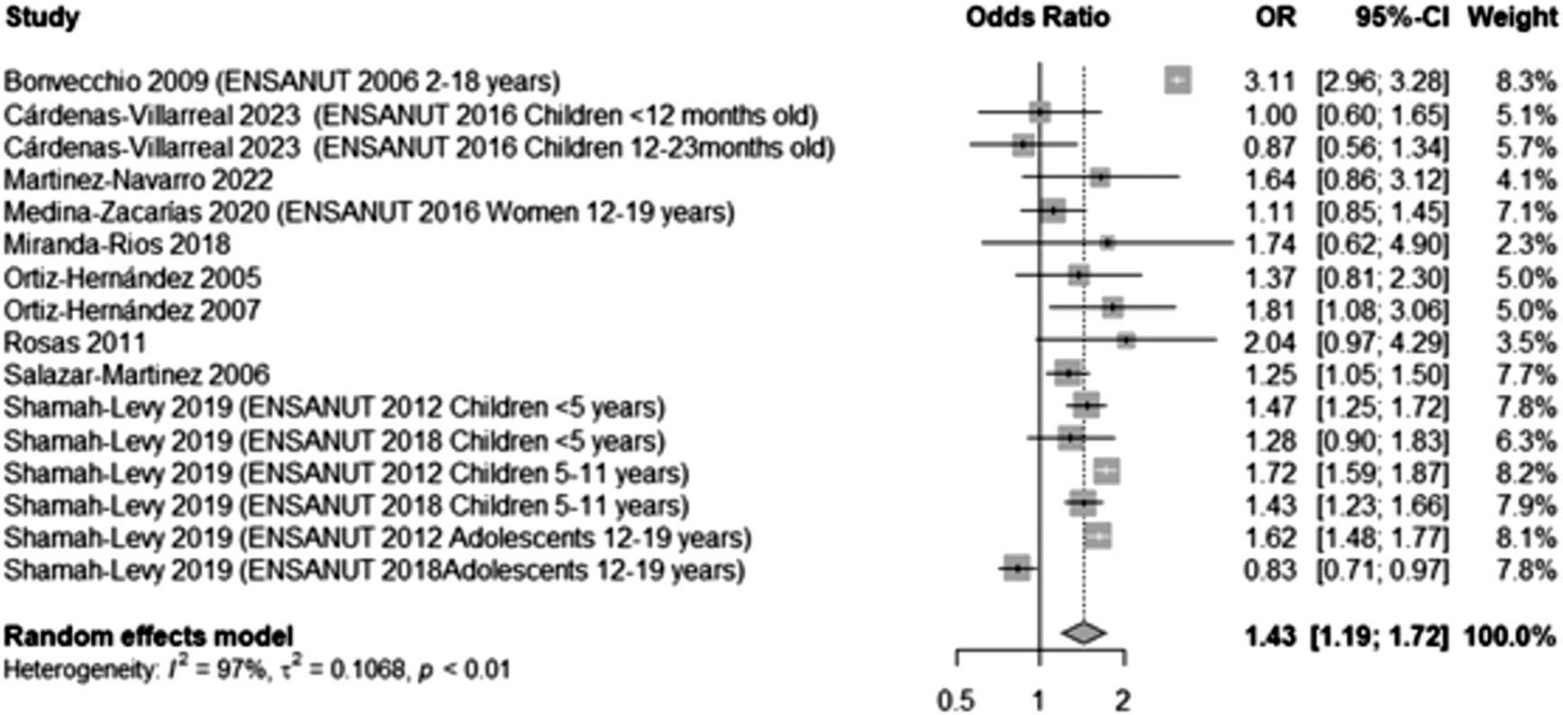
Likelihood of overweight and obesity in participants from wealthier households compared to those from poorer households. This analysis included data from 83,596 participants and compared those in the highest BMI categories (overweight and obesity) with those who had a normal BMI, excluding underweight participants whenever possible. This meta‐analysis pooled participants categorized as “medium” and “high” SES to highlight those with better household characteristics and compared them to those categorized as “low” SES (including those with the poorest household characteristics). Two studies by Miranda‐Rios (2018) and Ortiz‐Hernández (2005) used overcrowding (the number of people per room) as a proxy for wealth. In this analysis, Ortiz‐Hernández's 2007 measurement of household goods possession was included; the remaining studies utilized categories from principal component analysis, encompassing household assets and structure.

Ten studies^37^, ^41^, ^59^, ^60^, ^62^, ^65^, ^66^, ^72^, ^73^, ^75^, ^76^ that reported wealth measurements at a household level were meta‐analyzed. The meta‐analysis revealed that children and adolescents from wealthier households (with better characteristics and structure, classified as “medium” or “high” SES) were significantly more likely to have either overweight or obesity compared to those classified as having a low SES (OR 1.43, 95% CI 1.19, 1.72, Figure 2). This likelihood remained significant for children when accounting only for obesity (OR 1.47, 95% CI 1.08, 2.01, Appendix 3, Figure S1).

Also, when considering only those children with the highest wealth (i.e., the best household characteristics and structure, and removing those deemed to have a “medium” SES) compared to those with the lowest wealth categories (i.e., worst household characteristics and structure), the likelihood became higher, showing that those with the best household characteristics were significantly more likely to have either overweight or obesity (OR 1.65, 95% CI 1.46, 1.86, Appendix 3, Figure S2), a likelihood which was even higher when considering obesity only (OR 2.62, 95% CI 1.63, 4.19, Appendix 3, Figure S3).

These results remained significant even when analyzing them in light of the methods used to recall the measurement. For those studies measuring wealth through household goods, services, and structure, those children considered to live with better services/structure were likelier to have either overweight or obesity (OR 1.42, 95% CI 1.17, 1.74, Appendix 3, Figure S4), or obesity only (OR 1.69, 95% CI 1.22, 2.35, Appendix 3, Figure S5). [Correction added on 29 April 2025, after first online publication: The previous sentence has been corrected in this version.] For those studies using household overcrowding as a measurement of household wealth, a similar trend was found with those children living in not‐overcrowded households (as a proxy of better wealth) with a higher likelihood of having overweight or obesity (OR 1.59, 95% CI 1.12, 2.27, Appendix 3, Figure S6).

### 3.2 Living setting (urban vs rural areas)

Twenty‐seven studies considered the living environment, based on the size of the communities where participants lived, a proxy of SES.^27^, ^31^, ^32^, ^33^, ^34^, ^36^, ^37^, ^39^, ^40^, ^41^, ^43^, ^44^, ^45^, ^48^, ^49^, ^51^, ^54^, ^56^, ^57^, ^58^, ^60^, ^67^, ^69^, ^73^, ^74^, ^75^, ^76^, ^77^, ^78^, ^82^, ^85^. Generally, studies considered rural areas to be those with less than 2500 inhabitants. (Appendix 2) Overall, most of the studies showed higher prevalences of overweight and obesity in children living in urban settings. However, two studies^33^, ^34^, ^36^ found no statistically different prevalence of overweight or obesity among those children living in rural or urban areas. One of these studies, presented in two references, included only Indigenous children of Tarahumara origin.^33^, ^34^

**FIGURE 3:**
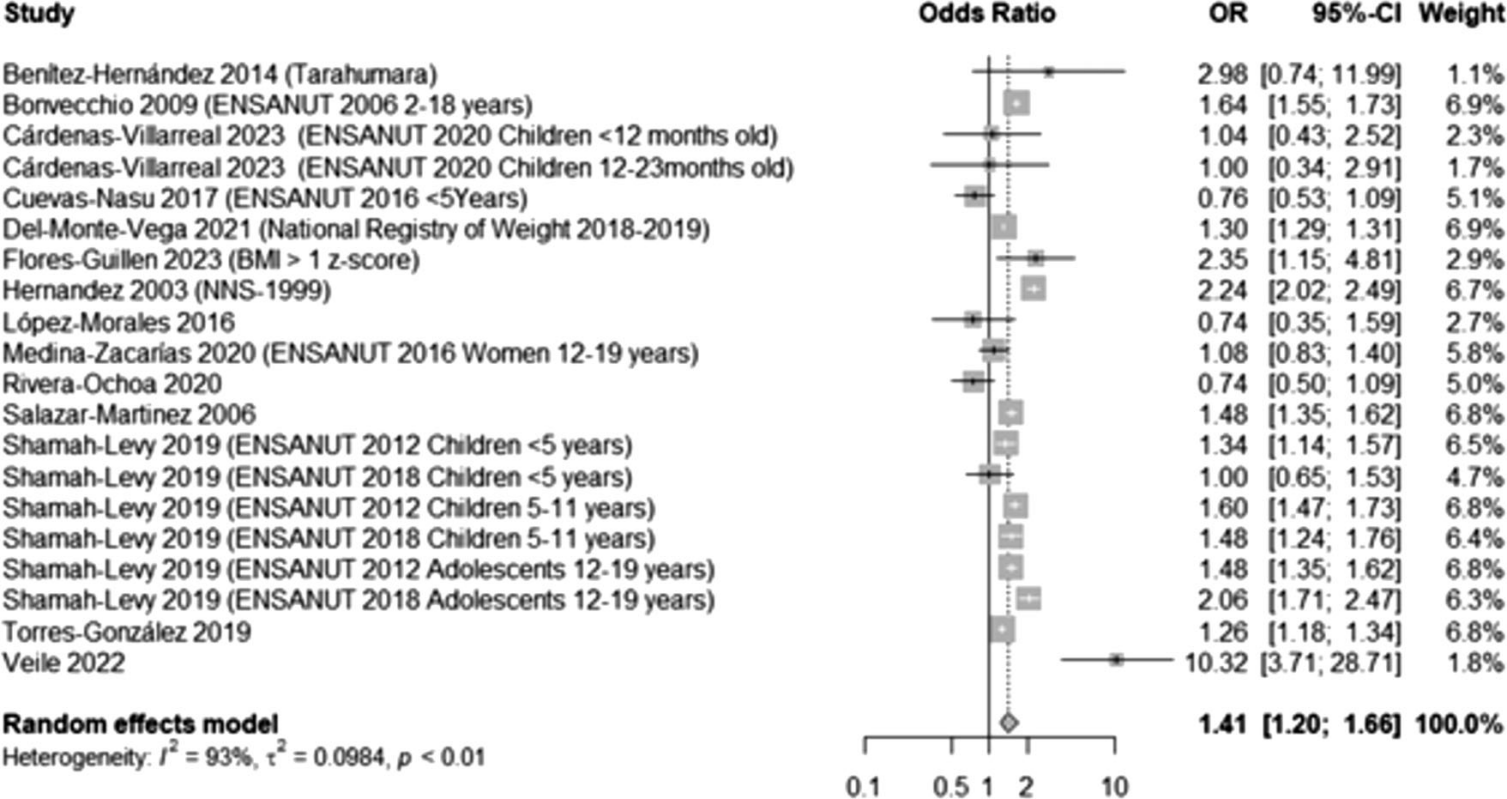
Likelihood of overweight and obesity in participants living in urban areas compared to those living in rural areas. This analysis included data from 2,647,633 participants and compared those in the highest BMI categories (overweight and obesity) with those who had a normal BMI, excluding underweight participants whenever possible. Rural areas were considered those areas with less than 2500 inhabitants.

Fourteen studies^33^, ^37^, ^41^, ^44^, ^45^, ^69^, ^73^, ^75^, ^76^, ^77^, ^82^ that provided sufficient data were meta‐analyzed. Results show that participants living in urban areas are significantly more likely to have overweight or obesity compared to those living in rural areas (OR 1.41, 95% CI 1.20, 1.66, Figure 3). This likelihood was higher and remained significant when considering obesity only (OR 1.64, 95% CI 1.39, 1.93, Appendix 3, Figure S7).

### 3.3 Parental education

Twenty‐four studies^32^, ^35^, ^36^, ^38^, ^39^, ^40^, ^42^, ^46^, ^47^, ^48^, ^51^, ^53^, ^54^, ^55^, ^58^, ^59^, ^62^, ^64^, ^67^, ^70^, ^72^, ^78^, ^80^, ^81^ considered parental education as a proxy of SES. However, four studies included this characteristic in a comprehensive measurement of SES, and no direct association with the weight status of children was provided^35^, ^38^, ^39^, ^67^. From the rest, 11 studies^32^, ^40^, ^46^, ^51^, ^55^, ^58^, ^59^, ^64^, ^70^, ^72^, ^81^ included maternal education, and the remaining studies included the education level of both parents.^36^, ^42^, ^47^, ^48^, ^53^, ^54^, ^62^, ^78^ One study examined maternal education by years of schooling, while paternal education was assessed based on overall literacy levels.^54^

Most of the studies reported higher prevalences of childhood and adolescent overweight or obesity among households with better‐educated parents compared to those with the least‐educated parents.^32^, ^40^, ^47^, ^48^, ^51^, ^54^, ^55^, ^58^, ^62^, ^70^, ^72^ However, a few studies also reported that children of mothers who were less educated or had lower literacy were likely to have overweight or obesity.^46^, ^53^, ^79^ One study found no difference in the nutritional status of offspring based on maternal education.^59^

One study^51^ reported a high correlation between maternal education and overall SES (wealth estimation). Ullman et al^78^ used data from a national sample and reported that higher paternal education was associated with a lower prevalence of obesity. Nonetheless, the association between maternal education and obesity was positive but not always significant (as it largely depended on other SES variables included in the adjusted analysis). Also, a study conducted among Mayan children^42^ showed that compared to the normal weight mother and child dyads, with each year increase of maternal education, there was a significant decrease in the odds of overweight and obesity in mothers and children. Conversely, with each year's increase in the father's education, there was also an increase in the odds of having overweight or obesity among fathers and children.

**FIGURE 4:**
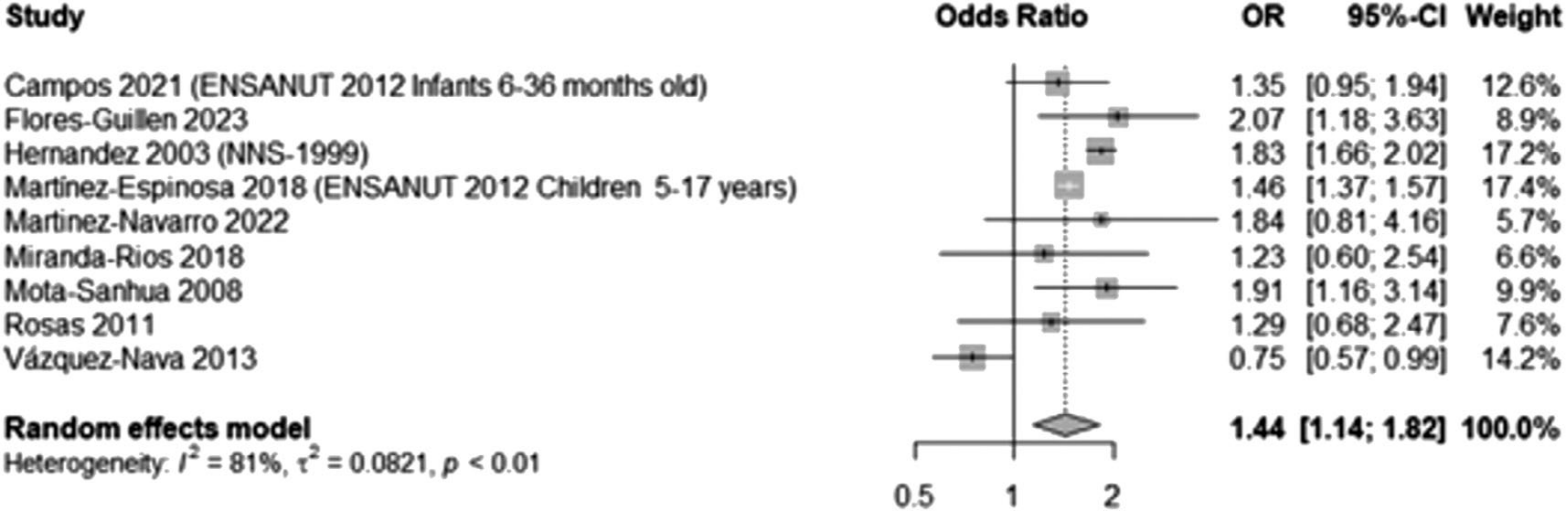
Likelihood of overweight and obesity in participants with well‐educated mothers compared to those with least‐educated mothers. This analysis included data from 29,981 participants and compared those in the highest BMI categories (overweight and obesity) with those who had a normal BMI, excluding underweight participants whenever possible. For this analysis, well‐educated mothers were defined as those with educational qualifications equivalent to secondary education or higher. They were compared to those with less‐educated mothers, defined as those with primary education or below. Flores 2019 was excluded because the “low education” category included individuals with less than a high school education, which did not align with the other studies that classified the least educated as those with six or fewer years of education (equivalent to primary school in Mexico). Additionally, two further studies were excluded as they reported using the same ENSANUT data as Martinez‐Espinosa 2018.

Ten studies^40^, ^48^, ^51^, ^54^, ^58^, ^59^, ^62^, ^64^, ^72^, ^81^ that provided sufficient data on maternal education were meta‐analyzed. Children and adolescents with well‐educated mothers (secondary school or higher) were significantly more likely to have overweight or obesity than those with the least educated mothers (primary/elementary studies or less) (OR 1.44, 95% CI 1.14, 1.82, Figure 4). This likelihood was even higher and remained significant when comparing participants with the most educated mothers (college studies or more) compared to those with the least educated mothers (primary/elementary studies or less) (OR 1.94, 95% CI 1.79, 2.10, Appendix 3, Figure S8). Due to insufficient data, meta‐analyses of paternal education were not possible.

### 3.4 Family structure and size

Eighteen studies^30^, ^35^, ^38^, ^39^, ^40^, ^42^, ^46^, ^56^, ^57^, ^58^, ^60^, ^62^, ^64^, ^68^, ^72^, ^78^, ^79^, ^81^ accounted for family size or structure as a proxy of SES. From these, three studies^38^, ^57^, ^78^ accounted for this characteristic as a covariate within their analysis, and no direct association with overweight and obesity was presented. Twelve studies^30^, ^35^, ^39^, ^40^, ^42^, ^46^, ^56^, ^58^, ^60^, ^62^, ^64^, ^68^ accounted for the number of people conforming to the household as a proxy of SES. Seven studies^35^, ^39^, ^42^, ^58^, ^62^, ^64^, ^68^ found that participants with the highest prevalences of overweight or obesity were found among smaller families, while two^30^, ^56^ reported higher prevalences of overweight or obesity among participants from larger families. Two other studies found^40^, ^46^ no difference in the prevalence of overweight or obesity according to the number of people in the house. However, one study found a significant likelihood of overweight and obesity only when accounting for a higher number of siblings but not with a higher number of people conforming to the family.^64^

Along with the number of family members, nine studies^40^, ^46^, ^56^, ^58^, ^62^, ^68^, ^72^, ^79^, ^81^ also considered parents' marital status or maternal partner status. Three studies^40^, ^46^, ^72^ found that the partner status, marital status, or father's absence of mothers were not predictors of overweight or obesity among participants. One^56^ reported a significant difference in the prevalence of obesity depending on the composition of the family, with a higher percentage of participants with obesity from families with no fathers. One abstract^79^ found that children from unmarried couples were more likely to have obesity than those from married couples. Two studies^58^, ^62^ found that children with single mothers were less likely to have overweight or obesity compared to those with both parents at home. One study^81^ found a higher prevalence of absence in the home of one or both biological parents among children with overweight or obesity. Two studies also considered adolescent participants and considered the marital status of these adolescents as a predictor of overweight and obesity among female participants,^38^, ^60^ and one of these^60^ found that those who cohabit with a partner report a higher prevalence of overweight but not obesity.

Five studies^39^, ^40^, ^58^, ^62^, ^68^ considered cohabitation with other family members (usually grandmothers) as a potential factor contributing to overweight or obesity. One^58^ described that children from parents or single mothers who cohabit with relatives were more likely to have overweight or obesity. One study^62^ found that children from nuclear families had a higher prevalence of overweight or obesity compared to those from other familial compositions, including extended families. However, one study found that cohabiting with grandparents does not differentiate the prevalence of overweight or obesity among participants.^40^ The rest did not report differences between cohabitation status and nutritional status.

### 3.5 Ethnic origin

Sixteen studies^32^, ^33^, ^36^, ^37^, ^40^, ^41^, ^42^, ^46^, ^48^, ^54^, ^58^, ^60^, ^61^, ^67^, ^76^, ^82^ considered the ethnic origin of participants as a proxy of SES. However, five^33^, ^42^, ^47^, ^67^, ^82^ recruited participants from specific ethnic groups but did not study the role of ethnicity in participants' overweight or obesity status. From those studies that recruited non‐Indigenous and Indigenous participants, seven studies^32^, ^36^, ^37^, ^41^, ^54^, ^60^, ^76^ recalled Indigenous status if at least one woman in the household or household head spoke an indigenous language. One study^40^ captured ethnicity by asking mothers if they self‐identified as Indigenous. One more^46^ used two variables to account for ethnicity, whether an indigenous language was spoken in the household or for the type of community (greater or not than 50% of the community is indigenous). One study^61^ recruited participants in Yucatan and recorded ethnicity through the last name of participants (having two Mayan surnames, one Mayan surname, or a non‐Mayan surname). Three studies^27^, ^50^, ^67^ also considered children from Indigenous schools, and two reported a lower prevalence of overweight and obesity for those participants attending Indigenous schools compared to general public and private schools.^27^, ^50^

Generally, the prevalence of overweight and obesity among non‐Indigenous participants was higher when compared to their counterparts.^37^, ^40^, ^54^, ^58^, ^76^ However, some studies only found higher prevalences in specific age or sex groups (e.g., women 11–19 years only, but not younger populations).^32^, ^60^, ^76^ Some others did report no differences in the prevalence of overweight and obesity according to the ethnic origin of participants.^36^, ^46^

**FIGURE 5:**
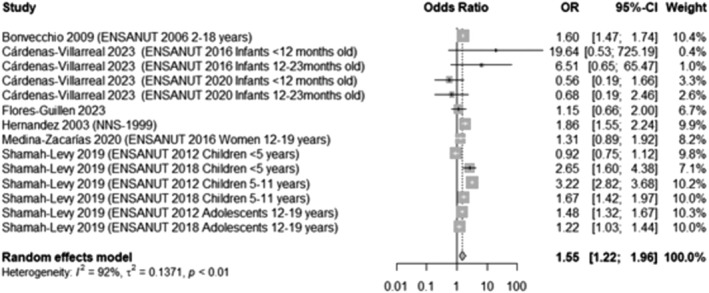
Likelihood of overweight and obesity in non‐Indigenous participants compared to Indigenous participants. This analysis included data from 83,344 participants and compared those in the highest BMI categories (overweight and obesity) with those who had a normal BMI, excluding underweight participants whenever possible. Ethnicity was recorded at a household level, with most of the studies recording if at least one woman or head of >12 years spoke an indigenous language and if it was the case, households were considered to be Indigenous.

A meta‐analysis was conducted, including six studies^37^, ^41^, ^48^, ^54^, ^60^, ^76^ that categorized their participants as Indigenous or non‐Indigenous. The results show that non‐Indigenous participants are significantly more likely to have overweight or obesity compared to Indigenous children (OR 1.55, 95% CI 1.22, 1.96, Figure 5).

This likelihood was higher when considering only obesity (OR 2.22, 95% CI 1.93, 2.56, Appendix 3, Figure S9).

### 3.6 Income or monetary measurements

Thirteen studies^27^, ^35^, ^36^, ^39^, ^52^, ^53^, ^55^, ^61^, ^62^, ^67^, ^68^, ^73^, ^80^ included household income or monetary measurements as a proxy of SES. However, half of these studies included income as part of a compound measurement or as a covariate within their analysis, and no direct association between income and overweight or obesity was presented.^27^, ^35^, ^36^, ^39^, ^62^, ^67^, ^73^ From the other half, income or monetary assets were measured differently. For example, three studies^55^, ^61^, ^80^ accounted for the total family income. One^52^ accounted for two indicator variables for medium and high‐income inequality: GDP per capita and schooling for each federal entity. Another study^53^ included monthly family income, spending on food per month (percentage of minimum wage), and expenses in food per capita per month (percentage of salary minimum). One study^68^ accounted for the perception of financial issues.

One study found that the prevalence of overweight or obesity at high inequality levels was higher than the low or medium inequality level participants. However, this was only significant for boys.^52^ Gonzalez‐Rico et al^53^ found that food expenditure per capita per month (percentage of monthly salary) was significantly associated with obesity. However, this was included as a covariate of a model measuring the association of family dysfunction with obesity. Mendez et al^61^ reported that the odds of having overweight or obesity were significantly lower among children from higher‐income families. Ramirez‐Serrano et al^68^ found that those children living with families that perceived having economic issues were significantly more likely to have overweight or obesity. On the contrary, Jimenez‐Cruz et al^55^ reported that children living in households with a monthly income >600 US dollars were more likely to have obesity. Due to the differences in the data and measuring methods of income or monetary measurements, meta‐analyses were not possible.

### 3.7 Parental occupation or employment

Twelve studies accounted for parental employment as a proxy of SES.^38^, ^39^, ^40^, ^53^, ^56^, ^57^, ^62^, ^64^, ^72^, ^80^ Three studies included this variable as a covariate within their analysis as part of an index or compound measurement, and no direct association between parental occupation and overweight or obesity was presented.^38^, ^53^, ^56^ The categorization of this factor varied widely across studies, from those reporting parents having or not having jobs to those such as Campos et al^40^ that conceptualized maternal employment as having a paid job (including the hours worked in a week) but also accounted for whether the job was formal (including a tax contributory social protection system) and if it was a full‐ or part‐time job.

Overall, six studies reported no statistical differences in the prevalence of overweight or obesity among children from working mothers compared to those with unemployed mothers.^39^, ^40^, ^62^, ^72^, ^80^, ^81^ Manila et al^57^ studied two cohorts of children in Oaxaca in 1972 and 2000. This study categorized SES by using only the working status of parents. They found that the prevalence of overweight and obesity did not differ among SES groups in 1972 and 2000; however, the prevalences were significantly higher within each SES group in 2000 compared to 1972.

**FIGURE 6:**
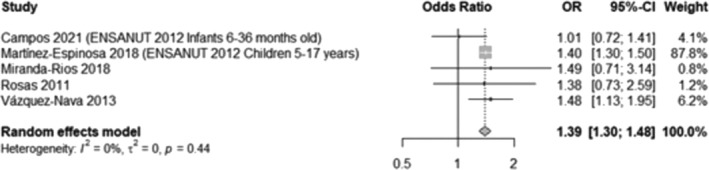
Likelihood of overweight and obesity in participants with working mothers compared to those with unemployed mothers. This analysis included data from 18,195 participants and compared those in the highest BMI categories (overweight and obesity) with those who had a normal BMI, excluding underweight participants whenever possible. Variables were dichotomized, considering whether mothers had any jobs or were unemployed. Mora‐Sanhua 2008 was excluded from the analysis as it was unclear if the measurement included only mothers or both parents.

A meta‐analysis was conducted, including five studies^40^, ^58^, ^62^, ^72^, ^81^ that evaluated maternal employment and overweight and obesity prevalence. We found that participants with employed mothers (any type of employment) are more likely to have overweight or obesity, compared to those with unemployed mothers (OR 1.39, 95% CI 1.30, 1.48, Figure 6). Due to insufficient data, the analysis only for obesity considering different types of employment (e.g., full‐time vs part‐time, or formal vs. informal), or considering fathers' employment was not possible.

### 3.8 Type of school

Eight studies^27^, ^28^, ^29^, ^50^, ^59^, ^70^, ^83^, ^84^ considered the type of school participants attended as a proxy of SES. Two study papers considered participants from different types of schools, including public (without a financial fee), private (with an economic fee), Indigenous (public schools located in rural communities with Indigenous populations monolingual and bilingual), and schools from the National Council of Educational Development (public schools located in small rural areas that also benefit the migrant community and have a maximum of 29 children each).^27^, ^50^ One paper also considered the type of public school (offering full‐time education, morning or afternoon shift).^27^ Five studies^27^, ^28^, ^29^, ^50^, ^84^ reported a higher prevalence of overweight and obesity among private schools. Nevertheless, two studies^59^, ^70^ showed a higher prevalence of overweight and obesity among children from public schools. One study found no differences in the prevalence of overweight and obesity, considering the different school types.^83^

**FIGURE 7:**
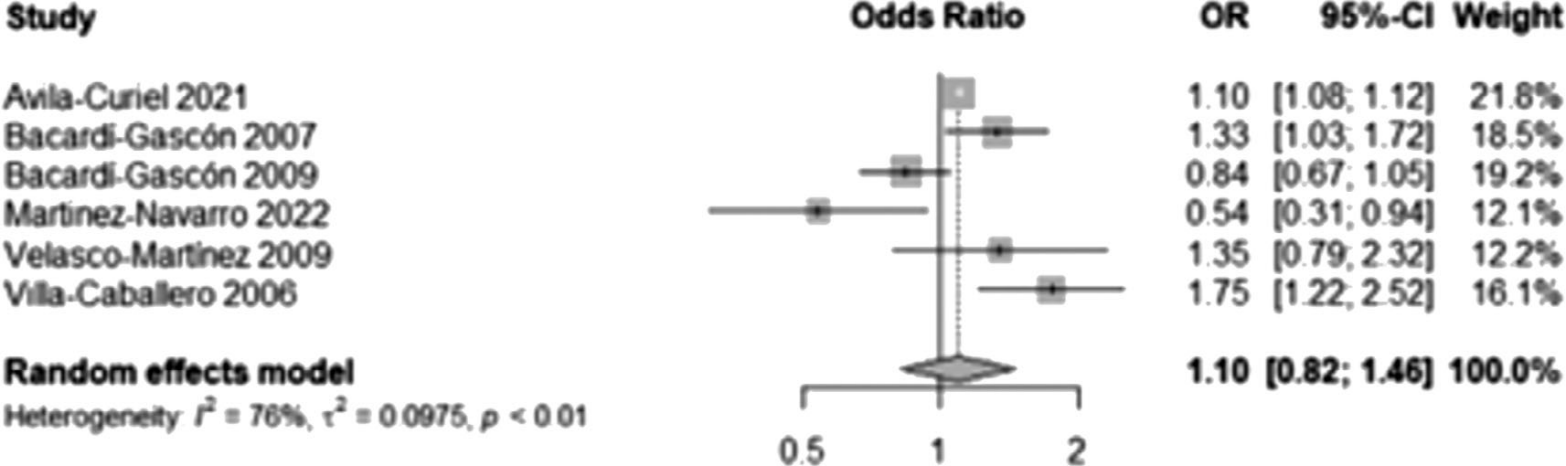
Likelihood of overweight and obesity in participants attending private schools compared to those attending public schools. This analysis included data from 10,532,924 participants and compared those in the highest BMI categories (overweight and obesity) with those who had a normal BMI, excluding underweight participants whenever possible. Public schools were those provided by the government without a financial fee, and private schools had an economic fee.

A meta‐analysis was conducted, including six studies^27^, ^28^, ^29^, ^59^, ^83^, ^84^ that provided information about public and private schools and the prevalence of overweight and obesity. We found no significantly higher likelihood of overweight and obesity among participants attending private schools than those attending public schools. (OR 1.10, 95% CI 0.82, 1.46, Figure 7).

According to one study,^27^ those participants assisting with an afternoon shift schooling were less likely to have overweight or obesity than those attending morning shifts or full‐time schooling. Moreover, one longitudinal study^38^ found that adolescents who finished only primary school are more likely to transition to obesity compared to adolescents who continue or finish secondary school.

### 3.9 Other factors identified as SES proxies in Mexico

As part of the ENSANUT survey, health service affiliation was considered a potential SES attribute.^60^, ^75^, ^76^ No statistical differences in the prevalence of overweight or obesity were reported for these characteristics.^60^ Also, ENSANUT records whether participants are food aid program recipients; however, no statistical differences in the prevalence of overweight or obesity were reported according to their participation in food programs.^43^, ^60^, ^75^, ^76^ Other studies that recorded food aid as an SES characteristic found similar results, such as Fernald et al^46^ which conducted a community survey and reported whether a federal breakfast program benefited the community. Such a study found no significantly higher likelihood of overweight or obesity among children in communities that received such programs. Morales‐Ruan et al^63^ reported in an abstract that no association existed between food aid and overweight or obesity. Two studies^38^, ^60^ recorded child/adolescent work as a potential SES indicator from participants, but no results were provided in relation to their nutritional status.

### 3.10 Risk of bias

Most of the studies covered all the items evaluated through the JBI tool. However, abstracts were the ones falling short of reporting key methodological details. From the items evaluated, the criteria for inclusion in the sample were clearly defined for most of the included papers, except for 11 papers (mainly abstracts),^33^, ^39^, ^48^, ^50^, ^52^, ^63^, ^70^, ^77^, ^79^, ^80^, ^85^ where this was unclear. Likewise, some details were ambiguous or not reported when describing the setting in 14 studies.^27^, ^33^, ^34^, ^35^, ^42^, ^49^, ^50^, ^52^, ^53^, ^56^, ^63^, ^71^, ^77^, ^79^, ^80^, ^85^ The methods to collect or categorize SES data within 11 studies were unclear.^45^, ^48^, ^50^, ^52^, ^53^, ^62^, ^63^, ^70^, ^71^, ^79^, ^80^, ^85^ Moreover, weight measurements to estimate overweight and obesity were mainly recollected using validated and reliable methods across all papers. Appendix 4 provides a complete overview of the evaluation of the risk of bias.

## 4 DISCUSSION

For the first time, the concept of “socioeconomic status” and its relation to overweight and obesity in children and adolescents was systematically reviewed within the Mexican context. Data from 54 studies indicated that the most frequently used SES proxy in research related to childhood obesity in Mexico was wealth based on household characteristics and asset possession, followed by living area (urban vs rural), parental education, family structure, ethnic origin, household income, parental occupation, type of school, health care access and food aid provisions. Our research revealed that Mexican children and adolescents were significantly more likely to have overweight or obesity if they were categorized as having higher wealth (estimated by household characteristics), lived in urban areas, identified as non‐Indigenous, had mothers with at least secondary school education or higher, or had mothers who were employed. Generally, in middle‐income countries, higher SES has been linked to obesity due to increased affluence, leading to dietary shifts toward higher‐energy, processed foods and reduced physical activity stemming from greater access to technology and less active transportation.^8^ While most proxies associated with obesity in Mexican children suggest a higher SES, not all indicators of higher SES—such as attending private school or not participating in a food provision program—were significantly linked to childhood overweight or obesity. Moreover, the evidence for other SES factors identified in this review, such as family structure, family size, household income, and financial measures, remained uncertain.

The relationship between SES and childhood obesity varies across high‐, middle‐, and low‐income countries.^8^ For instance, low maternal education is linked to childhood obesity in high‐income countries.^86^, ^87^ Nevertheless, Mexican children whose mothers have higher levels of education were found to have increased rates of overweight or obesity. Interestingly, this trend was similar to the one reported in Colombia, with children of mothers with higher education having significantly higher rates of overweight, even if this trend was not significant for fathers' education level.^88^ In contrast, in Brazil, maternal education was reported as insignificantly related to overweight, while children were reported to have significantly lower overweight rates if their fathers had some college education or higher.^88^ Parental education has been emphasized as a reliable proxy for SES among young people, as it is relatively stable and does not fluctuate due to transient life events such as employment or income. Although it does not directly impact the development of obesity in children, it influences behaviors, lifestyles, and other SES factors, such as perceived income and occupation.^89^, ^90^, ^91^ Nevertheless, the inconsistent relationship between parental education and childhood obesity in upper‐middle‐income Latin American countries highlights discrepancies among SES factors in similar nations. This suggests that other factors, such as cultural influences, are crucial for understanding the connection between SES and childhood obesity.

Mexican cultural values, beliefs, lifeways (especially food or food customs), and bonds with immediate and extended families, in particular female relatives, strongly influence childhood and adolescent obesity‐related lifestyles in Mexico.^17^ Mexican mothers are perceived by children and society as the primary caregivers responsible for feeding and nurturing families, emphasizing their crucial role in addressing obesity.^17^ Interestingly, most studies only consider the attributes of mothers when accounting for parental SES. Nevertheless, most of the evidence found in Mexican children relied on fixed, one‐dimensional SES indicators rather than adopting a more holistic approach that considered the complexity and cultural significance of SES variables, along with the dynamics of Mexican families. For example, participants with employed mothers were more likely to have overweight or obesity when compared to those with unemployed mothers. Yet, most studies simplified the parental employment indicator into a dichotomized variable (employed vs unemployed), overlooking the complexities of employment in Mexico. One of the included studies^40^ highlighted the multiple layers of “maternal employment” within the Mexican context, as it accounted for whether mothers had a job, whether the job was full‐ or part‐time, and whether the job was formal. The study found that 67.5% of mothers with children aged 6–35 months were unemployed.^40^ Although statistically insignificant, it suggested a slightly higher likelihood of overweight among children of part‐time working mothers (formal and informal) than those of full‐time working mothers when compared to non‐working mothers.^40^ Women are underrepresented in Mexico's labor force, with an estimated participation rate of 46.2% in 2023, compared to 76.2% for men.^92^ Approximately 56% of both men and women work in informal employment.^92^ Women's involvement in the Mexican informal economy has been marginal and largely involuntary, driven by necessity, family responsibilities, time and childcare constraints, limited human capital, and persistent socioeconomic disadvantages compared to men.^93^ In Mexico, job informality often refers to employers not being registered as businesses with tax authorities or jobs lacking the benefits and protections mandated by law, which can limit access to public healthcare to women and their offspring.^91^ Additionally, jobs in the informal economy are typically much lower paid than those in the formal sector, leaving some women unable to meet their children's basic needs.^93^ Also, Mexican women in informal employment experience higher rates of health problems, including obesity, which can also contribute to childhood obesity.^94^

In addition to parental characteristics, this review also identified certain factors related to living area and ethnicity that were accounted for within the SES concept in Mexico. The evidence showed that individuals living in urban areas or identified as non‐Indigenous were significantly more likely to experience overweight or obesity compared to their rural or Indigenous counterparts. In Mexico, rural and Indigenous communities share a strong connection, with different Indigenous groups traditionally residing in rural areas where they preserve their cultural practices, languages, and communal lifestyles, often relying on agriculture, crafts, and traditional livelihoods. However, not all urban areas are inhabited by Indigenous peoples, nor are all Indigenous communities rural. In 2020, 6% of the population aged 3 years and older was considered to speak an indigenous language, and 79% of the Mexican population lived in urban areas (>2500 people).^95^, ^96^ Evidence indicates significant disparities in economic resources, social services, infrastructure, healthcare access, and food environments between rural and urban areas in Mexico, resulting in varying childhood obesity rates.^69^ These inequalities are mainly caused by uneven development resulting from rapid urbanization and industrialization in larger cities.^97^ A higher affluence and transformation in general lifestyles among urban areas has contributed to the higher obesity rates in Mexico in recent decades, with larger cities offering greater access to a broader range of food retail options, including highly energy‐dense and more processed foods.^97^ Additionally, it has been reported that Mexican adolescents in rural areas are more physically active than their urban counterparts, a trend attributed to a more active lifestyle characterized by modes of active transportation, outdoor work, household chores, and fewer sedentary activities such as screen use time.^69^ Nevertheless, only a few studies examined the intersection and moderator/mediator effect of various SES factors and their association with obesity. For example, one included study explored the living environment and ethnicity and found no significant difference in obesity rates between Tarahumara participants residing in urban and rural areas.^33^

This review also showcased methodological challenges while measuring SES in Mexico. For instance, one of the included studies^66^ compared the prevalence of overweight using two proxies for household wealth: overcrowding and the number of goods at home. The results indicated a nearly significant difference in the prevalence of overweight when measured by overcrowding (p = 0.079), but not when measured by the number of goods at home (p = 0.766). This illustrates the complex and multidimensional nature of SES variables, where different SES variables may yield divergent results, which researchers can perceive and interpret differently due to the absence of standardization and harmonization. Additionally, many SES variables used in studies were self‐reported, introducing challenges such as subjectivity, accuracy, reliability, social desirability bias, missing or inaccurate data, and variability in interpretation.^98^ Especially when collecting SES data from parents, bias might also be introduced as parents might be reluctant to disclose sensitive information, fearing judgment or stigma.^99^

One^38^ of the included studies had a longitudinal design and could capture the dynamic nature of SES characteristics and follow participants from childhood into adolescence. Such work found that individual characteristics are less significant than family factors in the chances of developing overweight or obesity. Moreover, this study also reported that the transition of any family member toward obesity was more relevant in determining the transition to obesity among normal‐weight children than family SES (wealth, measuring household characteristics), emphasizing that SES is a dynamic measurement that might change over time. For children, longitudinal data is crucial for understanding how early‐life SES influences later health outcomes and provides a comprehensive view of SES over time, identifying key life stages for effective obesity prevention and long‐term effects of adverse SES conditions.^100^, ^101^ Although economic wealth plays a crucial role in influencing household characteristics, as well as access to nutritious food and leisure activities for Mexican children,^17^ it is not the sole factor contributing to the disadvantage of SES. The evidence presented by Brambila‐Paz et al^38^ highlights that family dynamics and characteristics are also essential when interpreting SES association with childhood obesity. Although this review did assess factors linked to family, evidence exposed considerable variability, as factors assessed within these categories were non‐standardized and, hence, challenging to synthesize. For instance, evidence regarding familial SES included the number of household members, the number of siblings, partner status, marital status, father's absence, and cohabitation with grandparents; however, the evidence was either weak or inconsistent in linking these factors to childhood obesity.

This review's strengths include being the first to conceptualize SES within the Mexican context. This work included an extensive search across several databases and one search engine in two languages, which helped us capture relevant publications. As part of the COMO project, a search for gray literature relevant to childhood obesity in Mexico was conducted, enabling us to revise gray literature references.^15^ However, none met the inclusion criteria of the current review. Moreover, we evaluated the quality of the papers using the JBI tool to ensure consistency in the appraisal process, enhance reliability, reduce bias, and make it less prone to skewed interpretations. Yet, some limitations involved the search strategy focusing on titles, abstracts, and keywords. We might have missed references that included SES components in their analysis but did not highlight them in the abstract. Additionally, this work does not differentiate between children and adolescents, as most of the evidence is presented to the broader group under 18 years old. These might be a limitation, as adolescent populations are much more autonomous and independent, and SES components that might be relevant to children might not be as relevant to adolescents. SES within this review was often relatively narrowly defined in terms of household and setting levels. It is becoming increasingly recognized that this oversimplification does not account for the individual experiences of children and adolescents.^102^, ^103^ Other limitations include the variability in SES measurements across studies, which can affect pooled estimates, and the cross‐sectional design of most studies, which limits the ability to infer causality.

This work presents evidence of childhood obesity inequalities in Mexico. Although addressing social determinants of health has been identified as a key strategy to reduce the burden of obesity and prevent its onset,^101^ SES conditions are challenging to change in Mexico, as there is a consistent social and health structural inequality.^104^ The current work highlights that wealth (estimated through household characteristics), living area, ethnicity, maternal education, and employment were significantly associated with obesity among Mexican children and adolescents. These factors are unevenly distributed among the Mexican population and can impact living conditions, access to resources and assets, and family dynamics, which may affect childhood obesity rates. Yet, the analysis of SES influences on childhood obesity would be incomplete if cultural factors, decisions, behaviors, perceptions, attitudes, and family dynamics inherent in Mexican culture are ignored. Family and food are central cultural values in Mexico, with a strong intergenerational influence on food and feeding practices among children, adolescents, and their families. Likewise, misconceptions persist among children and their relatives (for instance, believing that childhood overweight indicates healthiness), perceived societal issues (such as insecurity and limited opportunities for exercise), and perceived economic challenges (like the unaffordability of organized physical activities for children, such as team sports, which impose costs on families), which lead to obesity‐related lifestyles.^17^

Given the complex pathways and mechanisms affecting the prevalence and distribution of childhood obesity in Mexico, no single intervention can reverse the trends of the past decades. Mexico has already made some progress at a policy level by introducing the sugar‐sweetened beverage tax^105^ and the warning label nutrient profile on food products marketed^106^ to address broader social and environmental factors by advocating for healthy lifestyles and mindful food purchases. However, interventions aimed at treating^18^ or preventing^19^ childhood obesity in Mexico generally lack a multi‐level, multi‐sector, multi‐disciplinary approach, as well as not being culturally adapted. Additionally, SES factors are key determinants of childhood obesity in Mexico, and understanding the interaction of SES factors at individual, familial, and community levels is crucial for reducing childhood obesity inequalities in Mexico. Mapping the systems contributing to childhood obesity inequalities and understanding the impact of various determinants (including SES and culture) will assist in prioritizing actions and evaluating the feasibility of changes in Mexico's individual, family, community, and policy levels. Additionally, it is crucial to adapt interventions and programs by identifying individuals at higher risk of obesity and tailoring messages and materials to resonate with cultural practices and beliefs related to food and the customs associated with obesity. This approach should target individuals and their nuclear and extended families, while also addressing misconceptions, to effectively tackle childhood obesity in Mexico.

## CONFLICT OF INTEREST STATEMENT

MA‐M and CFMG have no conflict of interest to declare. YYGG received funding from Bonafont to present in a congress in 2016 and funding from Abbott's company to write two book chapters in 2020.

## Supporting information


**Appendix S1.** Search strategy.
**Appendix S2**. Full description of SES variables across included studies.
**Appendix S3.** Results presented by different BMI categories and SES variables.
**Figure S1.** Likelihood of obesity in participants from wealthier households compared to those from poorer households.
**Figure S2.** Likelihood of overweight and obesity in participants from the wealthiest households compared to those from poorer households.
**Figure S3.** Likelihood of obesity in participants from the wealthiest households compared to those from poorer households.
**Figure S4.** Likelihood of overweight and obesity in participants living with better household services/structure compared to those living with worse household services/structure.
**Figure S5.** Likelihood of obesity likelihood in participants living with the best household services/structure compared to those living with the worst household services/structure.
**Figure S6.** Likelihood of overweight and obesity in participants from non‐overcrowded households compared to those from overcrowded households.
**Figure S7.** Likelihood of obesity in participants living in urban areas compared to those living in rural areas.
**Figure S8.** Likelihood of overweight and obesity in participants with mothers with high degrees (college or over) compared to those mothers with primary studies or less
**Figure S9.** Likelihood of obesity in non‐Indigenous participants compared to Indigenous participants.
**Appendix S4.** JBI Risk of Bias Assessment.
