# Supplementary material for: Socioeconomic determinants of overweight and obesity among Mexican children and adolescents: Systematic review and meta‐analysis
Source: Obes Rev. 2025 Apr 10;26(8):e13926. doi: 10.1111/obr.13926 (PMC12246893; doi:10.1111/obr.13926)
Supplement: Supplementary file 1 — Appendix S1. Search strategy. Appendix S2. Full description of SES variables across included studies. Appendix S3. Results presented by different BMI categories and SES variables. Figure S1. Likelihood of obesity in participants from wealthier households compared to those from poorer households. Figure S2. Likelihood of overweight and obesity in participants from the wealthiest households compared to those from poorer households. Figure S3. Likelihood of obesity in participants from the wealthiest households compared to those from poorer households. Figure S4. Likelihood of overweight and obesity in participants living with better household services/structure compared to those living with worse household services/structure. Figure S5. Likelihood of obesity likelihood in participants living with the best household services/structure compared to those living with the worst household services/structure. Figure S6. Likelihood of overweight and obesity in participants from non‐overcrowded households compared to those from overcrowded households. Figure S7. Likelihood of obesity in participants living in urban areas compared to those living in rural areas. Figure S8. Likelihood of overweight and obesity in participants with mothers with high degrees (college or over) compared to those mothers with primary studies or less Figure S9. Likelihood of obesity in non‐Indigenous participants compared to Indigenous participants. Appendix S4. JBI Risk of Bias Assessment. [file OBR-26-e13926-s001.pdf]

Supporting Information Files

Title: Socioeconomic Determinants of Overweight and Obesity among Mexican Children and Adolescents: Systematic Review and Meta-analysis.

Authors: Magaly Aceves-Martins[1]*, Yareni Yunuen Gutierrez-Gómez[2], Carlos Francisco Moreno-García[3]

Author's affiliation:

1. The Rowett Institute of Nutrition and Health. University of Aberdeen, Foresterhill, Aberdeen, AB25 2ZD, UK
2. School of Medicine and Health Sciences, Tecnologico de Monterrey, Mexico
3. School of Computing, Robert Gordon University, Garthdee House, Garthdee Road, Aberdeen, AB10 7QB, Scotland, UK

Contact information: Magaly Aceves-Martins, The Rowett Institute of Nutrition and Health, University of Aberdeen, Foresterhill, Aberdeen, AB25 2ZD, UK. email: magaly.aceves@abdn.ac.uk

Appendix 1. Search strategy

#	Query
1	(child* or adolescent* or infant* or newborn* or juvenile or pediatrics or paediatrics or juvenile or youth or boy? or girl? or kid*).ab,hw,kf,kw,oi,sy,ti.
2	(obesity or obes* body weight or weight or childhood obesity or adolescent obesity or overweight or Pediatric Obesity or BMI or body mass or body mass index or adiposity or adipos* or waist circumference or nutritional status or malnutrition).ab,hw,kf,kw,oi,sy,ti.
3	(poverty or extreme poverty or child poverty or poverty level or poor or rich or disadvantage* marginalization or marginalized or socioeconomic* or socioeconomic factors or social status or income* or SES or income distribution or income inequality or household income or family income or income group or lowest income group or income or social inequality or economic inequality or income inequality or inequalit* or wealth inequality or wealth* or social class or disadvantaged or disadvantaged population or vulnerable population or Family Affluence).ab,hw,kf,kw,sy,ti.
4	(education or educa* or occupation or occupation* neighbourhood or neighborhood or household or rural or urban).ab,hw,kf,kw,sy,ti.
5	(food provision or food program* or social program or food supply).ab,hw,kf,kw,sy,ti.
6	3 or 4 or 5
7	1 and 2 and 6
8	(Mexico or Mexican or mexic*).ab,hw,ia,kf,kw,sy,ti.
9	7 and 8
10	remove duplicates from 9

This search was conducted in Medline and Embase. Adaptations from this search were used for further databases and in Spanish.

Appendix 2. Full description of SES variables across included studies

The grey-highlighted variables show SES variables recorded and reported as part of the participant's characteristics but were not included in the analysis of overweight or obesity as part of the studies.

Study ID	Wealth	Living setting	Parental education	Family structure and size	Ethnic origin	Income	Parental employment	Type of school	Other factors
**Ávila-Curiel 2021**	Participants degrees of marginalisation in 5 categories: 1) very low 2) low 3) medium 4) high 5) very high, according to the National Population Council 2015 classification, which includes socioeconomic variables such as education, housing, population distribution and monetary income.	Localities: Rural <2,500 inhabitants and Urban > 2,500 inhabitants.	NR	NR	NR	Factors accounted for to describe participants or to build a broader SES estimation. However, it is not included as a potential variable (alone) in the study of overweight or obesity.	NR	Type of school**.** 1) Public: without financial fee; 2) Private: with a financial fee; 3) Indigenous: preferably located in rural communities with indigenous population monolingual and bilingual, and 4) The National Council of Educational Development (CONAFE): located in small rural and dispersed towns with a maximum of 29 children. Furthermore, they benefit the migrant population residing in agricultural camps. School shift: 1) morning (8:00 a.m. to 12:30 p.m.), 2) evening (2:00 p.m. to 6:30 p.m.), and 3) time complete for students with a school stay prolonged (two times: 8:00 a.m. to 2:30 p.m. and 8:00 a.m. to 16:00 hours).	NR

**Bacardí-Gascón 2007**	NR	NR	NR	NR	NR	NR	NR	Accounted, but the authors provided no definition.	NR
**Bacardí-Gascón 2009**	NR	NR	NR	NR	NR	NR	NR	Accounted, but the authors provided no definition.	NR
**Basaldua 2008**	NR	NR	NR	Number of children in a family.	NR	NR	NR	NR	NR
**Batis 2020**	ENSNAUT Wealth categories were based on a well-being condition index estimated with principal component analysis, which included household characteristics (e.g., material on the floor, walls, and roof, the availability of the public sanitary sewer, water and electricity) and assets (e.g., motor vehicle, television, computer, and refrigerator)	Rural <2,500 inhabitants and Urban > 2,500 inhabitants.	Maternal education was classified as Low (0–6 years of schooling: primary school or less) Medium (7–12 years of schooling: secondary to high school) High (>12 years of schooling: more than high school).	NR	A household was classified as Indigenous if at least one woman >12 years old spoke an indigenous language.	NR	NR	NR	NR

**Benítez-Hernández 2014**	NR	There is no definition provided. The authors state that 50% were recruited in an urban setting and 50% in a rural setting.	NR	NR	Factors accounted for to describe participants or to build a broader SES estimation. However, it is not included as a potential variable (alone) in the study of overweight or obesity. Only Tarahumara Indians were recruited. Participants were recruited if their four grandparents of the participants belonged to the Tarahumara ethnicity, and their families spoke their indigenous language.	NR	NR	NR	NR

**Bernabeu-Justes 2019**	NR	NR	Factors accounted for to describe participants or to build a broader SES estimation. However, it is not included as a potential variable (alone) in the study of overweight or obesity.	Family size (determined by number of siblings) and number of caregivers (mom, dad, both or only one).	NR	Factors accounted for to describe participants or to build a broader SES estimation. However, it is not included as a potential variable (alone) in the study of overweight or obesity. The monthly income (sufficient or insufficient) considering per capita household income Council Day National Evaluation of Social Development Policy (CONEVAL) of the 2014 statistical annexe that considers total current income per capita in the United States Mexicans from 3,460 pesos per month	NR	NR	NR

**Bojorquez 2018**	NR	Rural <2,500 inhabitants and Urban > 2,500 inhabitants.	Paternal education as:<br>None<br>Elementary (1–6 years)<br>Secondary: (7–9 years)<br>High school (10–12 years)<br>College or graduate school	NR	Household member who spoke an indigenous language (an indicator of being a member of an Indigenous group)	Factors accounted for to describe participants or to build a broader SES estimation. However, it is not included as a potential variable (alone) in the study of overweight or obesity. Perceived SES was computed from questions about household income availability (e.g., “Do you have enough money at home to purchase the clothes you need?”) with response options of never/sometimes/almost always/always. Responses were entered into a principal component analysis, and the first component was used.	NR	NR	NR

**Bonvecchio 2009**	The living conditions index is a proxy for SES. It uses principal components factor analysis based on household characteristics (number of rooms, running water, WC, and construction materials) and assets. The index is further divided into tertiles to represent low, medium, and upper socioeconomic status (SES) tertiles.	Rural <2,500 inhabitants and Urban > 2,500 inhabitants.	NR	NR	Ethnicity/Indigenous: For the 1988 survey, households were considered indigenous if located in predominantly indigenous municipalities, defined as those in which at least 40% of inhabitants spoke an indigenous language. For the 1999 and 2006 surveys, households were defined as indigenous if at least one woman aged 12 to 49 spoke an indigenous language.	NR	NR	NR	NR
**Brambila-Paz 2022**	The relative SES of individuals and households considered five characteristics of the dwelling unit: 1. Number of rooms. 2. Source of water. 3. Sanitary service. 4. Trash collection. 5. Fuel. The authors identified any housing unit that scored positive in one or more of the above indicators as precarious.	NR	Factors accounted for to describe participants or to build a broader SES estimation. However, it is not included as a potential variable (alone) in the study of overweight or obesity.	Factors accounted for to describe participants or to build a broader SES estimation. However, it is not included as a potential variable (alone) in the study of overweight or obesity.	NR	NR	Factors accounted for to describe participants or to build a broader SES estimation. However, it is not included as a potential variable (alone) in the study of overweight or obesity.	NR	In the follow-up, adolescents who were working were recorded.

**Brewis 2003**	Composite household SES measurement based on a 6-point scale based on lower, medium, or higher income, higher or lower status profession of parents, and whether the family lived in an apartment or house and had more bedrooms than household members or not)	Factors accounted for to describe participants or to build a broader SES estimation. However, it is not included as a potential variable (alone) in the study of overweight or obesity. All participants were from an urban area.	Factors accounted for to describe participants or to build a broader SES estimation. However, it is not included as a potential variable (alone) in the study of overweight or obesity. Mothers' and fathers' education level and higher or lower status profession of parents.	In characteristics fully described, they were counted as employed or not in analysis.	NR	Factors accounted for to describe participants or to build a broader SES estimation. However, it is not included as a potential variable (alone) in the study of overweight or obesity. All participants were from an urban area.	All fathers were employed at the time of the study. Mothers' modal varied. Hence, the study considered whether the mother was working or at home full time. The profession was also part of the composite household SES.	NR	NR
**Campos 2021**	ENSNAUT Wealth categories were based on a well-being condition index estimated with principal component analysis, which included household characteristics (e.g., material on the floor, walls, and roof, the availability of the public sanitary sewer, water and electricity) and assets (e.g., motor vehicle, television, computer, and refrigerator)	Rural <2,500 inhabitants and Urban > 2,500 inhabitants.	For maternal education, women were asked which was the last year of formal education they had completed and were categorised into four groups.	NR	Ethnicity was captured by asking mothers whether they self-identified as Indigenous.	NR	Maternal employment was conceptualised considering data on having a paid job, the number of hours worked during the past week, and formality. Full-time employment was defined as working 40 hours per week, and formality was described as having a paid job with contributory social protection systems. Both employment status and formality were combined into a unique 5-category variable.	NR	NR

**Cárdenas-Villarreal 2023**	ENSNAUT Wealth categories were based on a well-being condition index estimated with principal component analysis, which included household characteristics (e.g., material on the floor, walls, and roof, the availability of the public sanitary sewer, water and electricity) and assets (e.g., motor vehicle, television, computer, and refrigerator)	Rural <2,500 inhabitants and Urban > 2,500 inhabitants.	NR	NR	The element of indigeneity was defined as present if any member of the child's family spoke an Indigenous language.	NR	NR	NR	NR
**Cauich-Viñas 2019**	NR	NR	Parental education levels were recorded in years and grouped into five categories: none, primary school, junior high school, senior high school, and professional degrees (university and technical education).	The total number of people living permanently in the house	Factors accounted for to describe participants or to build a broader SES estimation. However, it is not included as a potential variable (alone) in the study of overweight or obesity. All children were Mayan	NR	NR	NR	NR

**Cuevas-Nasu 2009**	SES index categories were based on a well-being condition index estimated with principal component analysis, which included household characteristics (e.g., material on the floor, walls, and roof, the availability of the public sanitary sewer, water and electricity) and assets (e.g., motor vehicle, television, computer, and refrigerator)	Rural <2,500 inhabitants and Urban > 2,500 inhabitants.	NR	NR	NR	NR	NR	NR	Enrolment in other social assistance programs, food supplies, Soup Kitchens, or receiving dietary Supplements (vitamins and minerals) and support from non-governmental organisations.
**Cuevas-Nasu 2017**	NR	Rural <2,500 inhabitants and Urban > 2,500 inhabitants	NR	NR	NR	NR	NR	NR	NR
**Del Monte-Vega 2021**	Municipal marginalisation: very high, high, medium, low and very low, using the 2015 classification of the National Population Council.	Yes	NR	NR	NR	NR	NR	NR	NR

**Fernald 2007**	A proxy measure of SES was generated using household assets (e.g., car, van, refrigerator, blender, etc.) and housing quality (e.g., roof, wall and floor, number of rooms, presence of indoor bathroom, etc.). Also, the study used a subjective social status measurement estimated by the mothers using the MacArthur Scale of Subjective Social Status.	NR	Education and level of "intelligence" (measured through proficiency in the vocabulary) of the mother.	Number of people in the household, whether the father was present in the household, mother's marital status, mother's BMI, mother's height	Whether an indigenous language was spoken at home. A community survey was also conducted, and several conditions of the communities where the participants lived were assessed. Four key variables were selected for inclusion in the analyses described here, whether most of the community was indigenous.	NR	NR	NR	A community survey was also conducted, and several conditions of the communities where the participants lived were assessed. Four key variables were selected for inclusion in the analyses described here, including the school breakfast program (whether the community was receiving the benefits of a federal breakfast program), food supplies distribution program (whether the community was receiving food packages including staples such as oil, rice beans etc.).
**Flores 2019**	NR	NR	Both parents' education recall as Less than High School, High School/GED, Some college, University or higher.	NR	NR	NR	NR	NR	NR

**Flores- Guillen 2023**	Factors accounted for to describe participants or to build a broader SES estimation. However, it is not included as a potential variable (alone) in the study of overweight or obesity. Househ old assets recall.	Geographical living area (rural/urban). It is not specified how these areas were distinguished within the study.	Years of schooling of adolescents and their mothers	NR	Factors accounted for to describe participants or to build a broader SES estimation. However, it is not included as a potential variable (alone) in the study of overweight or obesity. Ethnicity was captured, considering whether the mother speaks an indigenous language.	NR	NR	NR	Factors accounted for to describe participants or to build a broader SES estimation. However, it is not included as a potential variable (alone) in the study of overweight or obesity. Access to health services was measured.
**Flores-Huerta 2012**	NR	Rural <2,500 inhabitants and Urban > 2,500 inhabitants	NR	NR	NR	NR	NR	NR	NR
**Galvan 2011 (A)**	NR	NR	NR	NR	NR	NR	NR	Type of school: public (general and Indigenous) versus private schools	NR
**García-Chávez 2020**	SES is estimated as low, middle, and high based on an index constructed using factor analysis (a principal-component approach), with information on dwelling characteristics and possession of household goods.	Rural <2,500 inhabitants and Urban > 2,500 inhabitants	Mother's educational level classified as: None Elementary school, Middle school, High school, Bachelor or higher	NR	NR	NR	NR	NR	NR
**García-Guerra 2012 (A)**	NR	NR	NR	NR	NR	Two indicator variables for medium and high-income inequality, GDP per capita and schooling for each federal entity	NR	NR	NR

**González-Rico 2012**	NR	NR	Mother's educational level	NR	NR	Factors accounted for to describe participants or to build a broader SES estimation. However, it is not included as a potential variable (alone) in the study of overweight or obesity.	Factors accounted for to describe participants or to build a broader SES estimation. However, it is not included as a potential variable (alone) in the study of overweight or obesity.	NR	NR
**Hernandez 2003**	The SES index was constructed using principal component analysis, using variables such as flooring materials, potable water, and ownership of appliances such as radio, television, refrigerator, washing machine, and stove.	Rural <2,500 inhabitants and Urban > 2,500 inhabitants	Father's literacy status (literate or illiterate according to reading and writing ability), maternal schooling (none, primary, secondary, high school, and higher education)	NR	Indigenous ethnicity (12- to 49-year older woman speaking a native language),	NR	NR	NR	NR
**Jimenez-Cruz 2010**	NR	NR	Maternal years of education.	NR	NR	The analysis uses household income (dollars/month) as a covariate.	NR	NR	NR

**López-Morales 2016**	NR	The geographic area where the family resides is divided into rural and urban; however, the criterion for designing the areas is not clear.	NR	Family Integration according to the WHO: I. Marriage; II. Extension (birth of first child, birth of last child); III. Full extension (birth of last child); IV. Contraction (first child leaves home); V. Complete contraction (last child leaves home); and VI. Dissolution (death of first spouse); The family APGAR (adaptation, participation, gradient of personal resource, affection, and resources) was applied to distinguish the functionality of said instrument.	NR	NR	Factors accounted for to describe participants or to build a broader SES estimation. However, it is not included as a potential variable (alone) in the study of overweight or obesity.	NR	NR

**Malina 2009**	NR	The children's places of birth were classified as in the colony or city of Oaxaca, elsewhere in the state of Oaxaca, another state of Mexico, or another country.	NR	Factors accounted for to describe participants or to build a broader SES estimation. However, it is not included as a potential variable (alone) in the study of overweight or obesity. Household size recalled.	NR	NR	Parental occupation is classified into six categories:<br>0. Household activities<br>1. Agricultural worker (males), common labourer-day labourer (loaders, sweepers, etc.), paid domestic worker (females), and street vendor (both sexes).<br>2. Skilled workers (mason, carpenter, mechanic, etc.), factory workers, artisans/craftsmen and craftswomen<br>3. Self-employed, small business chauffeur/driver, and salesperson), largely in the service sector.<br>4. Employees: Government workers at all levels and employees in private establishments<br>5. Professionals (teachers, architects, lawyers, engineers, etc.) and business owners. This classification was converted to an estimate SES using the higher-ranking occupation in two-parent families and the rank of the sole occupation in single-parent families as follows: Low SES:	NR	NR

							categories 0 and 1 Low-middle SES: categories 2 and 3 Middle SES: categories 4 and 5.		
**Martínez-Espinosa 2018**	SES, as provided by the dataset used by the study.	Three categories were used in area of living areas: less than 2500 inhabitants, from 2500 to 100,000 inhabitants, and more than 100,000 inhabitants.	Maternal scholarly	Size of the household and habitats besides nuclear family.	An Indigenous person sharing the residence	NR	Maternal employment (besides domestic) - labour participation of the mother by asking about the development of work activities the week before the interview, in addition to considering those who responded that they did have a job, although they did not work. The mother's labour was categorised dichotomously: 1 corresponds to her doing extra-domestic work and 0 to the opposite.	NR	NR
**Martinez-Navarro 2022**	To evaluate the families' SES, information was obtained on the characteristics of the home and assets such as refrigerators, washing machines, heaters, etc. Principal component analysis was used, and based on the score obtained, the households were divided into SES tertiles (low, medium, and high).	NR	Maternal scholarly (secondary education or less, high school or technical school, and university or postgraduate studies)	NR	NR	NR	NR	Private vs public schools	NR

**Medina-Zacarías 2020**	Well-being condition index: Accounting for information on housing construction material, structure, and assets possession (e.g., car or electrical appliances). To obtain a single factor that summarised the variability of these characteristics, a principal components analysis was carried out and the factor that explained 49.3% of the total variability was retained, which was classified into tertiles (medium, low and high).	Rural <2,500 inhabitants and Urban > 2,500 inhabitants	NR	Information on marital status, including whether the adolescents were married or living in a common law union, was identified.	Indigenism was identified when the head of the household reported speaking an indigenous language.	NR	NR	NR	The survey identified if the households were beneficiaries of any food programme, the eligibility of the head of the household for health services was identified (Mexican Institute Adolescent occupations were classified according to the activities carried out: working women, students, study and work, household activities, home and work, other situations.
**Mendez 2016**	NR	NR	NR	NR	Ethnicity. Full names in Mexico are structured by a first name and two surnames (father's first surname followed by mother's first surname). Participants were considered to have two Mayan surnames, or only one Mayan surname and one non-Mayan surname, and they had no Mayan surname.	Income. The amount of money an individual earns per working day (eight hours). This information was collected from the school records and parent incomes.	NR	NR	NR

**Miranda-Rios 2017**	Crowding as a wealth indicator accounted for the number of people per room	NR	Paternal education	Family composition is classified as Nuclear or Expanded, composite or other. Marital status of parents classified as married or single mothers, Number of family members	NR	Factors accounted for to describe participants or to build a broader SES estimation. However, it is not included as a potential variable (alone) in the study of overweight or obesity. Monthly family income reported.	Paternal occupation, Maternal occupation, Paternal employment situation, Maternal employment situation	NR	NR
**Morales-Ruan 2015 (A)**	SES, but it is unclear how this was measured	NR	NR	NR	NR	NR	NR	NR	Beneficiary of the School Breakfasts Programme
**Mota-Sanhua 2008**	NR	NR	Factors accounted for to describe participants or to build a broader SES estimation. However, it is not included as a potential variable (alone) in the study of overweight or obesity. The education of the mother or caregiver was based on the highest level of education completed in the educational system.	The number of family members was defined by the number of people who lived in the adolescents' houses.<br>The number of siblings was obtained from the adolescent.	NR	NR	Occupation of the parent is classified as:<br>a) Upper stratum<br>b) Upper middle stratum<br>c) Lower middle stratum<br>d) Low stratum	NR	NR

**Ortiz-Hernández 2005**	The SES was evaluated through the level of overcrowding accounted as the number of people who lived in the home was divided by the number of rooms used for sleeping, then categorised as high SES (0.50 to 1.99 people per room); medium SES (2.00 to 2.90 people per room) and low SES (2.91 to 10.00 people per room)	NR	NR	NR	NR	NR	NR	NR	NR
**Ortiz-Hernández 2007**	The SES was evaluated through the level of overcrowding accounted as the number of people who lived in the home was divided by the number of rooms used for sleeping, then categorised as high SES (≤ 1.49 people per room), medium SES (1.50 to 2.49) and low SES (≥ 2.50). Also, assets possession in the home (then the number of assets was added, and the children were classified into three categories: high (five goods), medium (four) and low (≤ 3).	NR	NR	NR	NR	NR	NR	NR	NR

**Peña-Reyes 2010**	Indicators of community well-being as: % of households without drainage or toilet facilities % of households without electricity % of households without piped water services. % of households without floors % of households in which >2 people sleep per room.	An index of marginalisation (relative position in society) for each municipality based on national data was used as part of the indicator of the community well-being index.	Factors accounted for to describe participants or to build a broader SES estimation. However, it is not included as a potential variable (alone) in the study of overweight or obesity. The overall indicator of community well-being considered % of individuals 15+ years who are illiterate, % of the population 15+ years without a complete primary education.	NR	Factors accounted for to describe participants or to build a broader SES estimation. However, it is not included as a potential variable (alone) in the study of overweight or obesity. Participants recruited were from bilingual schools for Indigenous children.	Factors accounted for to describe participants or to build a broader SES estimation. However, it is not included as a potential variable (alone) in the study of overweight or obesity. The overall indicator of community well-being considered % of employed persons with income up to two minimum salaries.	NR	NR	NR
**Ramírez Serrano 2021**	Type of housing (owned, borrowed and rented).	NR	NR	Family size number of people living in the child's home. Kind of family – type of union in which the parents love (e.g. married, separated, etc/) Type of family home - relation in which at least one of the family members is related to the head of the household (e.g., nuclear, expanded, composite. Parent's relationship: perception of the treatment between the children's parents, which can be stable, unstable, or conflictive.	NR	Perception of the family economy at the fortnight's end (no problem, some problems and many problems).	NR	NR	NR

**Rivera-Ochoa 2020**	NR	Rural <2,500 inhabitants and Urban > 2,500 inhabitants	NR	NR	NR	NR	NR	NR	NR
**Romano 2012 (A)**	NR	NR	Mothers educational level	NR	NR	NR	NR	Public vs private schools	NR
**Romero-Velarde 2009 (A)**	Social class of schools and SES but unclear how it was measured.	NR	NR	NR	NR	NR	NR	NR	NR
**Rosas 2011**	Household assets are measured in component analysis.	NR	Mother's education	Mother's marital status: Married or living as a married	NR	NR	Mother's work status	NR	NR
**Salazar-Martinez 2006**	The SES was generated using principal components analysis and had three categories: high, middle and low. The variables used were housing characteristics, ownership of durable consumer goods and family income.	Rural <2,500 inhabitants and Urban > 2,500 inhabitants	NR	NR	NR	Factors accounted for to describe participants or to build a broader SES estimation. However, it is not included as a potential variable (alone) in the study of overweight or obesity. Family Income as part of a composite measure.	NR	NR	NR

**Shamah-Levy 2019**	ENSNAUT Wealth categories were based on a well-being condition index estimated with principal component analysis, which included household characteristics (e.g., material on the floor, walls, and roof, the availability of the public sanitary sewer, water and electricity) and assets (e.g., motor vehicle, television, computer, and refrigerator)	Rural <2,500 inhabitants and Urban > 2,500 inhabitants	NR	NR	Accounted for the language spoken by the mother	NR	NR	NR	Participants were categorised as food programme beneficiaries or not. Type of health services access.
**Torres-González 2019**	NR	Rural <2,500 inhabitants and Urban > 2,500 inhabitants	NR	NR	NR	NR	NR	NR	NR
**Ullmann 2011**	Housing quality, household assets, and asset ownership were used to measure household wealth. Four items—running water, sanitation, good-quality floors, and whether the household cooks with wood—were considered to create an index.	Rural <2,500 inhabitants and Urban > 2,500 inhabitants	Maternal and paternal education: Categorised into: Low (fewer than 6 years of completed schooling) Medium (6–11 years completed); and High (12 or more years completed)	Factors accounted for to describe participants or to build a broader SES estimation. However, it is not included as a potential variable (alone) in the study of overweight or obesity. Only considered adolescents cohabiting with both parents.	NR	NR	NR	NR	NR
**Vasquez-Garibay 2011 (A)**	NR	NR	Yes - Unclear how it was measured	Number of living children	NR	Monthly per capita food expenses and budget for housing rent and services (water, gas, electricity).	Yes - Unclear how it was measured	NR	NR

**Vázquez-Nava 2013**	NR	NR	The mother's educational level was determined according to the number of academic years attended.	The type of family is defined based on the presence of the biological father, the biological mother, or both biological parents in the children's home.	NR	NR	Maternal employment	NR	NR
**Veile 2022**	NR	Yes, the proportion was reported in each setting.	NR	NR	Factors accounted for to describe participants or to build a broader SES estimation. However, it is not included as a potential variable (alone) in the study of overweight or obesity. Only Maya children were included.	NR	NR	NR	NR
**Velasco-Martínez 2009**	NR	NR	NR	NR	NR	NR	NR	Yes. Different schools accounted.	NR
**Villa-Caballero 2006**	Neighbourhood income level as marginalisation measure.	NR	NR	NR	NR	NR	NR	Public schools and Private schools	NR
**Walker-Pacheco 2011 (A)**	NR	Urban Guadalajara and half of rural Tierra Nueva. But no further explanation was provided.	NR	NR	NR	NR	NR	NR	NR

Socioeconomic Status (SES), National Nutrition and Health Survey (ENSANUT), Council Day National Evaluation of Social Development Policy (CONEVAL), Gross Domestic Product (GDP). SES factor not reported in the study (NR).

Appendix 3. Results presented by different BMI categories and SES variables

Supplementary Figure 1. Likelihood of obesity in participants from wealthier households compared to those from poorer households.

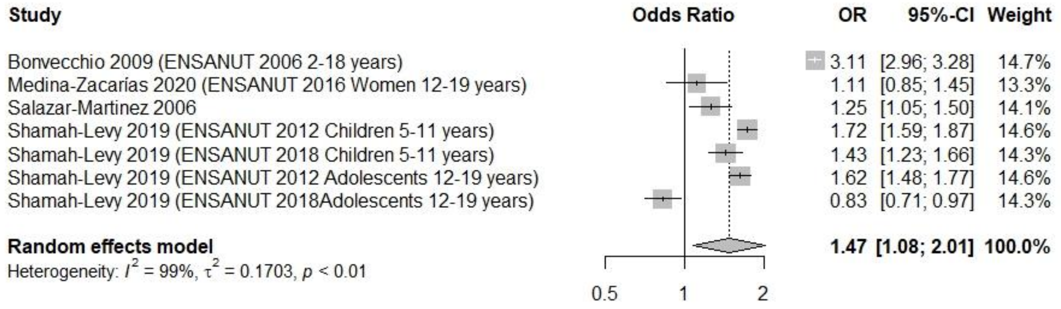

This analysis included data from 70,879 participants and compared those with obesity to those with a normal BMI, excluding underweight participants whenever possible. This meta-analysis pooled "medium" and "high" SES to highlight those with better household characteristics and compared them to those categorised as "low" SES (including those with the poorest household characteristics). All of the studies use categories of principal component analysis, including household assets and structure.

Supplementary Figure 2. Likelihood of overweight and obesity in participants from the wealthiest households compared to those from poorer households

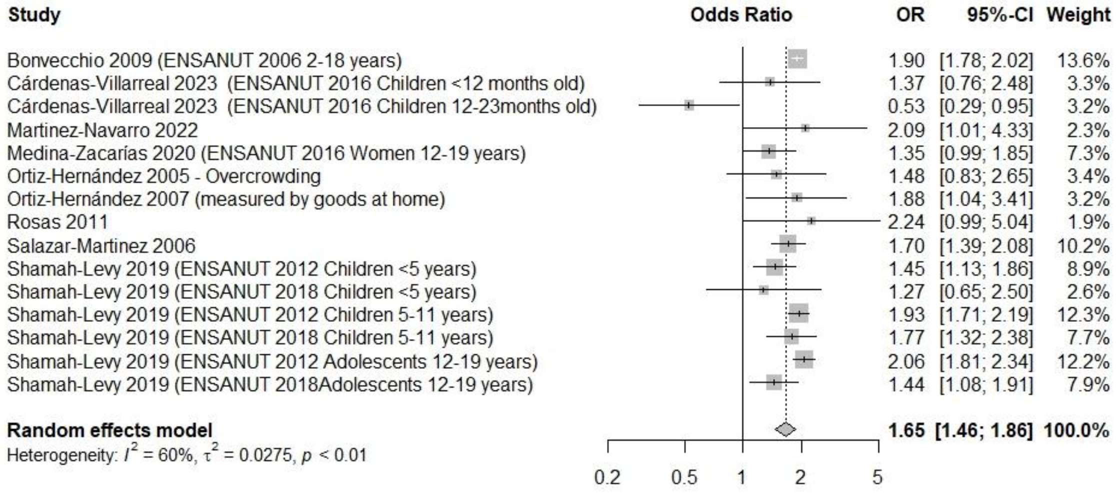

This analysis included data from 52,136 participants and compared those in the highest BMI categories (overweight and obesity) with those who had a normal BMI, excluding underweight participants whenever possible. This meta-analysis compared those classified as "high" SES (including the "better household characteristics" group) vs those categorised as "low" SES (including those with the worst household characteristics). All of the studies use categories of principal component analysis, including household assets and structure. Ortiz-Hernández (2005) uses overcrowding count as a proxy for wealth.

Supplementary Figure 3. Likelihood of obesity in participants from the wealthiest households compared to those from poorer households

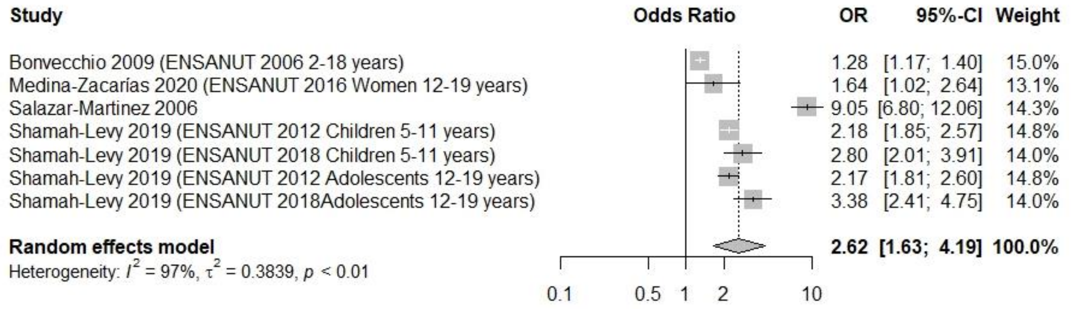

This analysis included data from 43,203 participants and compared those with obesity to those with a normal BMI, excluding underweight participants whenever possible. This meta-analysis compared those classified as "high" SES (including the "better household characteristics" group) vs those categorised as "low" SES (including those with the worst household characteristics). All of the studies use categories of principal component analysis.

Supplementary Figure 4. Likelihood of overweight and obesity in participants living with better household services/structure compared to those living with worse household services/structure

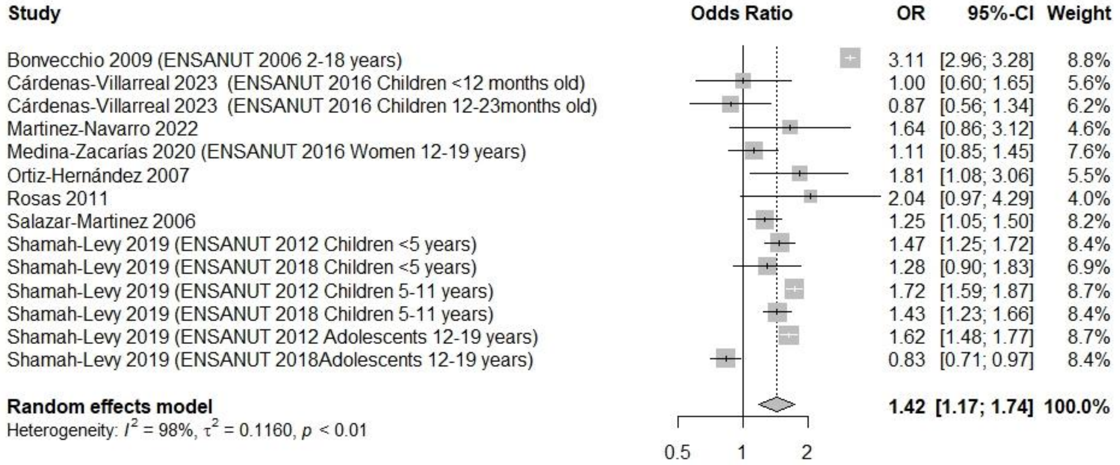

This analysis included data from 82,452 participants and compared those in the highest BMI categories (overweight and obesity) with those who had a normal BMI, excluding underweight participants whenever possible. This meta-analysis compared "high" SES (including the "better household characteristics" group) vs those categorised "low" SES (including those with the worst household characteristics). All of the studies use categories of principal component analysis, including household assets and structure.

Supplementary Figure 5. Likelihood of obesity likelihood in participants living with the best household services/structure compared to those living with the worst household services/structure

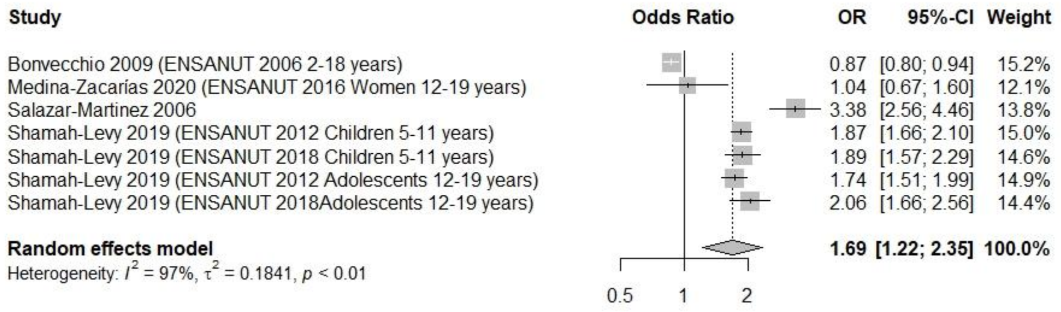

This analysis included data from 70,879 participants and compared those in the highest BMI categories (overweight and obesity) with those who had a normal BMI, excluding underweight participants whenever possible. This meta-analysis compared participants categorised as "high" SES (including the "better household characteristics" group) vs those categorised as "low" SES (including those with the worst household characteristics). All of the studies use categories of principal component analysis, including household assets and structure.

Supplementary Figure 6. Likelihood of overweight and obesity in participants from non-overcrowded households compared to those from overcrowded households.

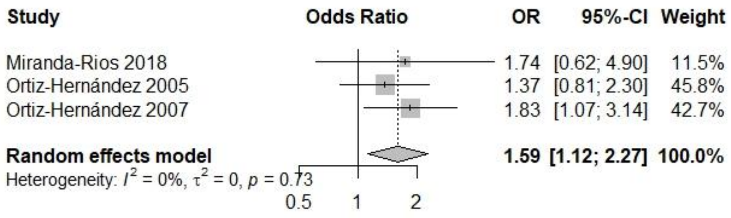

This analysis included data from 1,908 596 participants and compared those in the highest BMI categories (overweight and obesity) with those who had a normal BMI, excluding underweight participants whenever possible. This meta-analysis compared "medium" and "high" SES (including households considered not overcrowded) vs. those categorised "low" SES (including overcrowded households).

Supplementary Figure 7. Likelihood of obesity in participants living in urban areas compared to those living in rural areas

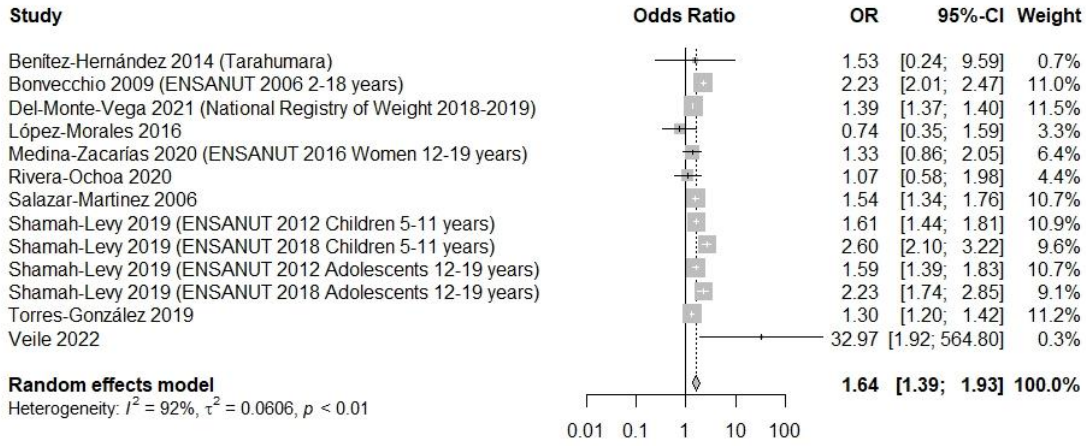

This analysis included data from 2,624,367 participants and compared those with obesity to those with a normal BMI, excluding underweight participants whenever possible. Rural areas were considered those areas with less than 2,500 inhabitants.

Supplementary Figure 8. Likelihood of overweight and obesity in participants with mothers with high degrees (college or over) compared to those mothers with primary studies or less.

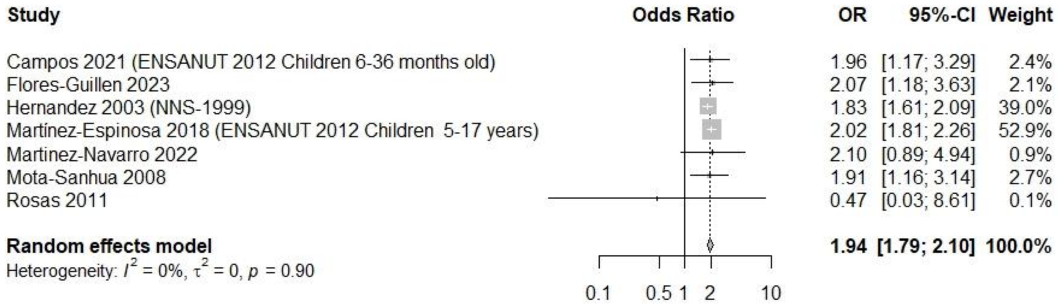

This analysis included data from 18,654 participants and compared those in the highest BMI categories (overweight and obesity) with those who had a normal BMI, excluding underweight participants whenever possible. For this analysis, well-educated mothers were defined as those with educational qualifications equivalent to college studies compared to those with least-educated mothers with educational qualifications equivalent to primary studies or less. Flores 2019was excluded the "low education" category, including those with less than high school, which did not align with the rest of the studies that considered the least educated as those with six or fewer years of education (equivalent to primary school in Mexico). Also, two studies were excluded as they reported using the same ENSANUT data as Martinez-Espinosa.

Supplementary Figure 9. Likelihood of obesity in non-Indigenous participants compared to Indigenous participants

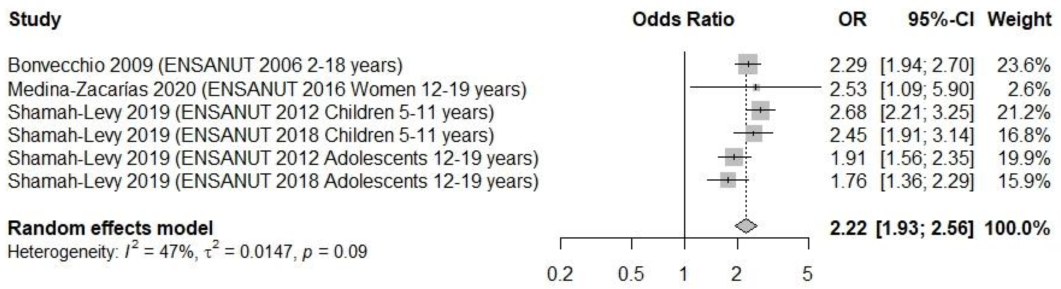

This analysis included data from 61,364 participants and compared those with obesity compared to those who had a normal BMI, excluding underweight participants whenever possible. Ethnicity was recorded at a household level, with most of the studies recording if at least one woman or head of >12 years spoke an indigenous language and if it was the case, households were considered to be Indigenous.

Appendix 4. JBI Risk of Bias Assessment

Study ID	***1. Were the criteria for inclusion in the sample clearly defined?***	***2. Were the study subjects and the setting described in detail?***	***3. Was the exposure measured in a valid and reliable way?***	***4. Were objective, standard criteria used for measurement of the condition?***	***5. Were confounding factors identified?***	***6. Were strategies to deal with confounding factors stated?***	***7. Were the outcomes measured in a valid and reliable way?***	***8. Was appropriate statistical analysis used?***
Ávila-Curiel 2021	Yes	Unclear	Yes	Yes	Yes	Yes	Yes	Yes
Bacardí-Gascón 2007	Yes	Yes	Yes	Yes	Yes	Yes	Yes	Yes
Bacardí-Gascón 2009	Yes	Yes	Yes	Yes	Yes	Yes	Yes	Yes
Basaldua 2008	Yes	Yes	Yes	Yes	Yes	Yes	Yes	Yes
Batis 2020	Yes	Yes	Yes	Yes	Yes	Yes	Yes	Yes
Benítez-Hernández 2014	Unclear	Unclear	Yes	Yes	Unclear	Yes	Yes	Yes
Bernabeu-Justes 2019	Yes	Unclear	Yes	Yes	No	No	Yes	Yes
Bojorquez 2018	Yes	Yes	Yes	Yes	Yes	Yes	No	Yes
Bonvecchio 2009	Yes	Yes	Yes	Yes	Yes	Yes	Yes	Yes
Brambila-Paz 2022	Yes	Yes	Yes	Yes	Yes	Yes	Yes	Yes
Brewis 2003	Unclear	Yes	Yes	Yes	Yes	Yes	Yes	Yes
Campos 2021	Yes	Yes	Yes	Yes	Yes	Yes	Yes	Yes
Cárdenas-Villarreal 2023	Yes	Yes	Yes	Yes	Yes	Yes	Yes	Yes
Cauich-Viñas 2019	Yes	No	Yes	Yes	Yes	Yes	Yes	Yes
Cuevas-Nasu 2009	Yes	Yes	Yes	Yes	Yes	Yes	Yes	Yes
Cuevas-Nasu 2017	Yes	Yes	Yes	Yes	Yes	Yes	Yes	Yes
Del Monte-Vega 2021	Yes	Yes	Unclear	Yes	Yes	Yes	Yes	Yes

Fernald 2007	Yes	Yes	Yes	Yes	Yes	Yes	Yes	Yes
Flores 2019	Yes	Yes	Yes	Yes	Yes	Yes	Yes	Yes
Flores-Guillen 2023	Unclear	Yes	Unclear	Yes	Yes	Yes	Yes	Yes
Flores-Huerta 2012	Yes	Unclear	Yes	Yes	Yes	Yes	Yes	Yes
Galvan 2011 (A)	Unclear	Unclear	Unclear	Unclear	Unclear	Unclear	Unclear	Unclear
García-Chávez 2020	Yes	Yes	Yes	Yes	Yes	Yes	Yes	Yes
García-Guerra 2012 (A)	Unclear	Unclear	Unclear	Unclear	Unclear	Unclear	Unclear	Unclear
González-Rico 2012	Yes	Unclear	Unclear	Unclear	Unclear	Yes	Yes	Unclear
Hernandez 2003	Yes	Yes	Yes	Yes	Yes	Yes	Yes	Yes
Jimenez-Cruz 2010	Yes	Yes	Yes	Yes	Yes	Yes	Yes	Yes
López-Morales 2016	Yes	No	Yes	Yes	Unclear	No	Yes	Unclear
Malina 2009	Yes	Yes	Yes	Yes	Yes	Yes	Yes	Yes
Martínez-Espinosa 2018	Yes	Yes	Yes	Yes	Yes	Yes	Yes	Yes
Martinez-Navarro 2022	Yes	Yes	Yes	Yes	Yes	Yes	Yes	Yes
Medina-Zacarías 2020	Yes	Yes	Yes	Yes	Yes	Yes	Yes	Yes
Mendez 2016	Yes	Yes	Yes	Yes	Yes	Yes	Yes	Yes
Miranda-Rios 2017	Yes	Yes	Unclear	Yes	Yes	Yes	Yes	Yes
Morales-Ruan 2015 (A)	Unclear	Unclear	Unclear	Unclear	Unclear	Unclear	Unclear	Unclear
Mota-Sanhua 2008	Yes	Yes	Yes	Yes	Yes	Yes	Yes	Yes
Ortiz-Hernández 2005	Yes	Yes	Yes	Yes	Yes	Yes	Yes	Yes
Ortiz-Hernández 2007	Yes	Yes	Yes	Yes	Yes	Yes	Unclear	Yes
Peña-Reyes 2010	Yes	Yes	Yes	Yes	Yes	Yes	Yes	Yes

Ramírez Serrano 2021	Yes	Yes	Yes	Yes	Yes	Yes	Yes	Yes
Rivera-Ochoa 2020	Yes	Yes	Yes	Yes	Yes	Yes	Yes	Yes
Romano 2012 (A)	Unclear	Yes	Unclear	Unclear	Unclear	Unclear	Unclear	Unclear
Romero-Velarde 2009 (A)	Unclear	Unclear	Unclear	Unclear	Unclear	Unclear	Unclear	Unclear
Rosas 2011	Yes	Yes	Yes	Yes	Yes	Yes	Yes	Yes
Salazar-Martinez 2006	Yes	Yes	Yes	Yes	Yes	Yes	Yes	Yes
Shamah-Levy 2019	Yes	Yes	Yes	Yes	Yes	Yes	Yes	Yes
Torres-González 2019	Unclear	Unclear	Yes	Yes	Unclear	Yes	Yes	Unclear
Ullmann 2011	Yes	Yes	Yes	Yes	Yes	Yes	Yes	Yes
Vasquez-Garibay 2011 (A)	Unclear	Unclear	Unclear	Unclear	Unclear	Unclear	Unclear	Unclear
Vázquez-Nava 2013	Yes	Yes	Yes	Yes	Yes	Yes	Yes	Yes
Veile 2022	Yes	Yes	Yes	Yes	Yes	Yes	Yes	Yes
Velasco-Martínez 2009	Yes	Yes	Yes	Yes	Yes	Yes	Yes	Yes
Villa-Caballero 2006	Yes	Yes	Yes	Yes	Yes	Yes	Yes	Yes
Walker-Pacheco 2011 (A)	Unclear	Unclear	Unclear	Yes	Unclear	Unclear	Unclear	Unclear
